# Clorgyline Analogs Synergize with Azoles against Drug Efflux in *Candida auris*

**DOI:** 10.3390/jof9060663

**Published:** 2023-06-13

**Authors:** Stephanie Toepfer, Michaela Lackner, Mikhail V. Keniya, Lisa-Maria Zenz, Marianne Friemert, Franz Bracher, Brian C. Monk

**Affiliations:** 1 Sir John Walsh Research Institute, Faculty of Dentistry, University of Otago, Dunedin 9016, New Zealandmikhail.keniya@hmh-cdi.org (M.V.K.); 2 Institute of Hygiene and Medical Microbiology, Medical University of Innsbruck, 6020 Innsbruck, Austria; michaela.lackner@i-med.ac.at (M.L.); lisa-maria.zenz@i-med.ac.at (L.-M.Z.); 3 Hackensack Meridian Health Center for Discovery and Innovation, Nutley, NJ 07110, USA; 4 Center for Drug Research, Department of Pharmacy, Ludwig-Maximilian University of Munich, 81377 Munich, Germany; marianne.friemert@cup.lmu.de (M.F.); franz.bracher@cup.lmu.de (F.B.)

**Keywords:** *Candida auris*, combination therapy, checkerboard assay, yeast pathogen, novel therapy, *Saccharomyces cerevisiae*, efflux pumps, Cdr1, Mdr1, antifungal, heterologous expression system, multidrug resistance

## Abstract

Concern about the global emergence of multidrug-resistant fungal pathogens led us to explore the use of combination therapy to combat azole resistance in *Candida auris*. Clorgyline had previously been shown to be a multi-target inhibitor of Cdr1 and Mdr1 efflux pumps of *Candida albicans* and *Candida glabrata*. A screen for antifungal sensitizers among synthetic analogs of Clorgyline detected interactions with the *C. auris* efflux pump azole substrates Posaconazole and Voriconazole. Of six Clorgyline analogs, M19 and M25 were identified as potential sensitizers of azole resistance. M19 and M25 were found to act synergistically with azoles against resistant *C. auris* clade I isolates and recombinant *Saccharomyces cerevisiae* strains overexpressing *C. auris* efflux pumps. Nile Red assays with the recombinant strains showed M19 and M25 inhibited the activity of Cdr1 and Mdr1 efflux pumps that are known to play key roles in azole resistance in *C. auris* clades I, III, and IV. While Clorgyline, M19 and M25 uncoupled the Oligomycin-sensitive ATPase activity of Cdr1 from *C. albicans* and *C. auris*, their mode of action is yet to be fully elucidated. The experimental combinations described herein provides a starting point to combat azole resistance dominated by overexpression of CauCdr1 in *C. auris* clades I and IV and CauMdr1 in *C. auris* clade III.

## 1. Introduction

The emergence of multidrug-resistant fungal pathogens is a global concern. Invasive candidiasis is estimated to affect 750,000 patients annually [1], with intrinsic and acquired resistance limiting therapeutic options. Increased administration of azole drugs to treat infections or as prophylaxis has exacerbated this situation. A US Centers for Disease Control and Prevention (CDC) report in 2017, which noted 1700 deaths were due to drug-resistant *Candida*, contributed to classification of these pathogens as a “serious health threat” [2]. The emerging fungal pathogen *Candida auris* includes members that are is resistant to multiple classes of antifungals [3,4,5,6]. The finding that 90% of *C. auris* clinical isolates were resistant to at least one antifungal class and 30% resistant to two or more contributed to its categorization as an “urgent health threat” [2]. Since its discovery 2009 [7], *C. auris* has been responsible for several outbreaks in hospitals and healthcare facilities worldwide [3,8,9,10,11,12,13,14]. *C. auris* is readily transmissible and often misdiagnosed and its adherence to surfaces makes it difficult to eradicate [15,16]. The World Health Organization (WHO) has recently prioritized new research into diagnosis, outbreak management, and treatment of *C. auris* [17].

Whole genome sequencing of *C. auris* revealed five clades: clade I (South Asia), clade II (East Asia), clade III (South Africa), clade IV (South America), and clade V (Iran) [3,18,19]. Current understanding of *C. auris* resistance patterns indicates, similar to *Candida albicans*, that selective low-level azole resistance is due to point mutations in the *ERG11 (CYP51)* gene. In *C. albicans*, the substitutions K143R and Y132H contribute to Fluconazole (FLC) resistance, while the substitution Y132F was reported to confer resistance to the triazole drugs except Itraconazole (ITC) and Posaconazole (POS) [20,21,22]. Point mutations homologous to these in *C. albicans* have been found in specific associations with *C. auris* clades: CauErg11 Y132F is found in clades I, IV, and V; K143R in clades I and IV; and F126L substitution is found exclusively in clade III [3,19,23]. Instances of the CauErg11 K143R mutation have occasionally been found in clade II isolates [24]. Recombinant *Sacharomyces cerevisiae* strains expressing CauErg11 Y132 and CauErg11 K143R showed elevated minimum inhibitory concentration (MIC) values for FLC and Voriconazole (VRC) but not POS when compared to wild-type CauErg11 [25]. Gain-of-function (GOF) mutations in the transcriptional factor Upc2 up-regulate ergosterol biosynthesis genes in *C. albicans*, leading to Erg11 overexpression and FLC resistance [26]. While GOF mutations in Upc2 have yet to be found in *C. auris*, copy number variation of *ERG11* due to gene duplication led to overexpression of CauErg11 [27].

GOF mutations causing overexpression of genes encoding the drug efflux pumps belonging to ATP-binding cassette (ABC) and major facilitator superfamily (MFS) contribute to high- and intermediate-level azole resistance, respectively, in *C. albicans*. The ABC efflux pumps Cdr1 and Cdr2 (*Candida* drug resistance 1 and 2) play key roles in azole resistance, conferring triazole resistance in *C. albicans* clinical isolates [28,29,30], with Cdr1 having the dominant role [31]. GOF mutations in the zinc-finger transcriptional factor Tac1, which enable constitutive overexpression of *CDR1*, are thought to be the principal drivers of CaCdr1 overexpression and high-level azole resistance. Triazole pan-resistant *C. albicans* clinical isolates overexpress *CDR1*, whereas GOF mutations in the zinc finger transcription factor Mrr1 cause overexpression of the MFS pump *MDR1* gene that enable resistance to short-tailed azole drugs, such as FLC and VRC, but not ITC or POS [32,33,34]. Furthermore, exposure to azole drugs can induce overexpression of *C. albicans* Erg11, Cdr1, and Mdr1 [35,36], possibly due to transcription factor binding exemplified by Pdr1 in *S. cerevisiae* [37].

Homologs of *C. albicans CDR1* and *MDR1* can be highly expressed in *C. auris*, with *CauCDR1* being the main contributor to triazole resistance [38,39,40]. As expected, CauCdr1 and CauMdr1 overexpressed in *S. cerevisiae* showed increased MICs to FLC and VRC, with Cdr1 overexpression also conferring increased MICs to POS and ITC [25]. The point mutation V704L in Cdr1 was found in azole-resistant isolates, whereas the amino acid substitutions A640V in Tac1b (clade I) and N647T in Mrr1 have been found in FLC resistant isolates (clade III) [4,38,39,41].

Echinocandin resistance in *C. auris* is linked to the S639F/P amino acid substitutions in the *FKS1* gene that are equivalent to S645F in *C. albicans FKS1* hot spot 1 [42,43,44]. Compared to FLC resistance, the occurrence of echinocandin resistance is still rare [42,44].

The low incidence of resistance to echinocandins has made them the first-choice treatment of *C. auris* infections, with the polyene Amphotericin B (AMB) used as an alternative therapy [45]. Unfortunately, therapeutic failure for both echinocandins and polyenes [3,6,42,44,46] and acquired resistance during echinocandin treatment have been reported [43]. Isolates resistant to azoles and echinocandins are defined as cross-resistant. Isolates resistant to azoles, echinocandins, and polyenes are defined as pan-resistant.

While the incidence of pan-resistant *C. auris* infection is still low, its emergence highlights the need for novel treatment strategies. In addition to developing novel antifungals, combinations of two antifungal drugs or an antifungal with a non-antifungal modulator have been tested in efforts to detect synergies that improve antifungal efficacy [47,48]. Recent examples include FLC with Ponatinib (PON) [49], Omeprazole (OME) [50], Rabeprazole (RAB) [50], or Clorgyline (CLO) [51]. A combination of ITC and Ospemifene (OSP) [52] was also tested. Additional examples of compounds that synergize with azole drugs are summarized in several reviews [53,54,55,56,57].

There are few examples of in vitro studies of multidrug-resistant isolates of *C. auris* where antifungal drug combinations [58,59,60,61,62] or antifungal/modulator combinations [52,63,64,65,66,67,68] were tested. Holmes et al. [51] found the monoamine oxidase inhibitor CLO inhibits *C. albicans* and *Candida glabrata* drug efflux pumps. We used agarose diffusion assays to screen the in vitro activity of POS, VRC, and Isavuconazole (ISA) alone and in combination with CLO and a range of CLO analogs and then characterized two hits as inhibitors of *C. auris* drug efflux. Recombinant *S. cerevisiae* strains overexpressing CauMdr1 (Y2765) and CauCdr1 (Y2766) [25] were used to determine how the hits interfere with individual mechanisms of drug efflux pump-mediated azole resistance prior to checkerboard assays with representative *C. auris* clade I and clade II clinical isolates.

## 2. Materials and Methods

### 2.1. Strains and Media

The strains used in this study are summarized in Table 1. *C. auris* strain AR0389 was obtained from the CDC & FDA Antimicrobial Resistance (AR) Isolate Bank (kindly provided by Professor David Perlin). *C. auris* strains CBS10913^T^ and CBS12875 were a gift from the Westerdijk Fungal Biodiversity Institute (Utrecht, Netherlands). The *C. albicans* clinical isolates TL1 and TL3 were kindly gifted by Professor Theodore White. *C. albicans* SGY-243 was kindly provided from the Squibb Institute for Medical Research, Princeton, NJ, USA and its FLC resistant mutant strain FR2 (isolated from SGY-243) was also obtained from this laboratory [28].

SD medium (Formedium, Norfolk, UK) containing 2% glucose, 10mM MES, and 20 mM HEPES buffered to pH 6.8 with TRIS was used for precultures, agarose diffusion plates (cultures solidified with 0.6% agarose), and assays with drug targets expressed in recombinant *S. cerevisiae* strains. Clinical isolates of *C. auris* and *C. albicans* were tested using RPMI1640 (Sigma-Aldrich, St. Louis, MO, USA) medium containing 2% glucose.

### 2.2. Chemicals

POS, VRC, FLC, ITC, AMB, Anidulafungin (ANA), Micafungin (MFG), Nile Red, 2-deoxy-D-glucose (2-DOG), OME, Rabeprazole sodium (RAB), and Oligomycin (OM) were purchased from Sigma-Aldrich (St. Louis, MO, USA). OSP, CLO, Enniatin B (ENB), and Beauvericin (BEA) were purchased from Cayman Chemical (Ann Arbor, MI, USA). PON was purchased from Sellecheck Chemicals LLC (Houston, TX, USA) and VT-1161 was synthesized by MicroCombiChem (Wiesbaden, Germany). ISA was purchased from BOC Sciences (Shirley, NY, USA). The CLO analogs M1-M25 were designed, synthesized [73,74], purified, and their structures confirmed using nuclear magnetic resonance (NMR), infrared spectroscopy (IR), and mass spectrometry (MS) (Supplemental Materials). The prodrugs OME and RAB were activated at low pH prior to the assay. OME was activated in SD-medium pH 3.5 for ten minutes. The assay was carried out at same pH as the active form of the compound is unstable at higher pH [75]. RAB is stable at higher pH and was activated for 1 h in the presence of 0.1 M HCl before diluting it into SD-medium with final pH 6.8 [76].

### 2.3. Agarose Diffusion Assay

Agarose diffusion assays were performed as previously described [77]. In brief, 5 mm paper disks (Whatmann, Buckinghamshire, UK) were loaded with 5 µL antifungal for the inhibition assay and/or 5 µL modulator for the combination assay and placed on SD pH 6.8 or RPMI1640 growth media solidified with 0.6% UltraPure^TM^ agarose (Invitrogen, Thermo Fisher Scientific, Waltham, MA, USA) containing *S. cerevisiae* or clinical isolates of *C. auris* inoculated at OD_600nm_ = 0.08. Control disks contained the same amount of DMSO. Zones of inhibition were measured after incubation for 48 h at 30 °C.

### 2.4. Antifungal Susceptibility Testing

Antifungal susceptibility testing of clinical isolates was performed according to the European Committee on Antimicrobial Susceptibility Testing (EUCAST) of Yeasts version 7.3.2. [78]. MIC values were obtained at ≥50% (MIC_50_) growth inhibition for azoles and echinocandin and ≥90% (MIC_90_) growth inhibition for AMB compared to the no drug control.

The susceptibility testing method was adapted for recombinant strains to allow optimal growth responses. SD medium buffered to pH 6.8 was used instead of RPMI1640, and the inoculum adjusted to OD_600nm_ = 0.01. MIC values were read in a Synergy 2 plate reader (BioTek, Winooski, VT, USA) and determined at ≥80% (MIC_80_) growth inhibition after correction for medium background.

### 2.5. Checkerboard Assay

This assay was performed as described previously [51]. Ninety six-well plates (Greiner Bio-One Cellstar, Kremsmünster, Austria) containing a 100 µL concentration gradients in medium combining antifungal and/or modulator were inoculated with 100 µL yeast (OD_600nm_ = 0.02) and incubated at 30 °C with shaking at 200 rpm (Minitron, Infors AG, Bottmingen, Switzerland) for 48 h. Growth was measured at OD_600nm_ using a Synergy 2 plate reader and corrected for medium background. Drug interactions were evaluated based on the Loewe additivity-based fractional inhibitory concentration index (FICI) model [79,80] using cutoffs suggested by Odds [81].

Fractional inhibitory concentration (FIC) at ≥80% growth inhibition and FIC indices (FICI) were calculated using following formulae:

$$FIC\;Agent\;A\;(antifungal)=\frac{{MIC}_{80\;Agent\;A\;in\;combination}}{{MIC}_{80\;Agent\;A\;alone}}$$

$$FIC\;Agent\;B\;(modulator)=\frac{{MIC}_{80\;Agent\;B\;in\;combination}}{{MIC}_{80\;of\;Agent\;B\;alone}}$$

FICI=FIC Agent A+FIC Agent B

According to guidelines, FICI data were interpreted as ‘synergy’ (FICI ≤ 0.5), ‘no interaction’ (FICI > 0.5–4.0), and ‘antagonism’ (FICI > 4.0) [81].

If the MIC_80_ for a compound could not be determined due to low toxicity, the next highest (double) concentration was used in calculating the FIC (e.g., MIC_80_ >25 mg/L = 50 mg/L).

### 2.6. Nile Red Assay

The Nile Red efflux pump assay was performed as described previously [25,77,82]. Cells (grown to OD_600nm_ = 2) starved for 30 min at 30 °C with 2-DOG (5mM final concentration) were loaded with Nile Red (7.5 µM final concentration). After incubation for 30 min at 30 °C, the cells were washed with HEPES and adjusted to OD_600nm_ = 10 in HEPES buffer, and 100 µL transferred to 96-well plates containing 50 µL inhibitor or HEPES buffer for the controls. Efflux at 30 °C was initiated by the addition of 50 µL 80 mM glucose to a final volume of 200 µL. Fluorescence intensity with an excitation wavelength 485/20 nm and an emission wavelength of 528/20 nm was measured every 35 sec for 10 min using a Synergy 2 plate reader.

### 2.7. ATPase Activity Assay

ATPase activity was determined as previously described [25,83,84,85]. Crude membranes (obtained by cell breakage and differential centrifugation) of recombinant strains were co-incubated with inhibitor in three-fold serial dilution in assay buffer. The reaction was stopped after 1 h incubation at 30 °C. The amount of free inorganic phosphate (Pi) was measured at 750 nm using Synergy 2 plate reader with KH_2_PO_4_ as standard.

## 3. Results

### 3.1. Antifungal Susceptibilities of C. auris Clade I and II Clinical Isolates

As there are no established MIC breakpoints for *C. auris,* tentative breakpoints defined by the CDC based on closely related *Candida* species were used [86]. These breakpoints (in mg/L) are as follows: FLC ≥ 32.0, ANA ≥ 4.0, MFG ≥ 4.0, and AMB ≥ 2.0. Breakpoints for the azoles ITC, VRC, POS, and ISA are not available, but the CDC suggests considering FLC susceptibility as a surrogate. The clinical isolate used was the type strain CBS10913^T^ (clade II), which is susceptible to all antifungal drug classes [7]. The azole resistant clinical isolates used were the clade I CBS12875 and AR0389 strains, both of which carry the Erg11 Y132F amino acid substitution. The AR0389 strain has also been shown to overexpress CauCdr1 several-fold compared to the clade I reference strain B8441 [40]. CBS12875 and AR0389 are >32- and >250-fold resistant to FLC and VRC, respectively, compared with CBS10913^T^ (Table 2). The MIC for ITC was ≥15-fold higher for the clade I strains compared with CBS10913^T^. CBS12875 showed a slightly higher MIC for POS than CBS10913^T^ (0.03 versus 0.06 mg/L), while AR0389 had a POS MIC (0.25 mg/L) 8-fold greater than CBS10913^T^. All strains were susceptible to the tested echinocandins ANA and MFG and AMB, with the exception of CBS12875 that was resistant to AMB (Table 2). The resistance profiles shown in Table 2 are consistent with both CBS12875 and AR0389 expressing CauErg11 Y132F, with AR0389 overexpressing CauCdr1 at 2 to 4-fold the level of CBS12875. These data indicate the phenotypic complexity of *C. auris* strains within clade I.

### 3.2. Screening CLO Analogs for Drug Efflux Modulation Using Disk Diffusion

CLO and its six analogs (Figure S1) were screened as modulators of antifungal azole activity using agarose diffusion assays at 50 nmol/disk. The compounds were tested using recombinant *S. cerevisiae* strains overexpressing CaMdr1A (Y525) or CaCdr1B (Y570) and clinicals isolates of *C. auris* CBS10913^T^ (clade II) and AR0389 (clade I). We considered values >1 as indicating synergy for the ratio defined as the inhibition zone radius for each azole drug in combination with a test compound divided by the inhibition zone for each azole drug alone (Table S1).

In the absence of an antifungal drug, CLO produced a small zone of inhibition but only with the control strain ADΔΔ. Both *N*-(cyclopropylmethyl)-3-(2,4-dichlorophenoxy)-*N*-methylpropan-1-amine (M19) and 3-(2,4-dichlorophenoxy)-*N*-methyl-*N*-(oxiran-2-ylmethyl)propan-1-amine (M20) produced a small zone of inhibition with the recombinant *S. cerevisiae* strains CaMdr1A and CaCdr1B. None of the other analogs gave a zone of inhibition.

Strain CaMdr1A functionally overexpresses the *C. albicans* MFS transporter Mdr1 that effluxes VRC and FLC strongly but POS weakly [70,77]. CLO (50 nmol/disk) combined with POS (0.05 nmol/disk), VRC (0.75 nmol/disk), or FLC (50 nmol/disk) strongly increased the size of the zone of inhibition. The combination of these azole drugs with the CLO analogs (50 nmol/disk) showed similar results as CLO, with some exceptions. Compound 2-((3-(2,4-dichlorophenoxy)propyl)(methyl)amino)acetonitrile (M18) in combination with FLC and M19 or M20 in combination with POS or VRC failed to increase the size of the inhibition zone.

Strain CaCdr1B functionally overexpresses the *C. albicans* ABC transporter Cdr1B that confers resistance to POS and VRC and extremely high resistance to FLC [70]. When tested with either VRC, POS, or FLC alone, no inhibition zone was detected. The combination of CLO (50 nmol/disk) with POS (45 nmol/disk) or VRC (12.5 nmol/disk), but not FLC (50 nmol/disk), gave zones of inhibition. The CLO analogs (50 nmol/disk) gave zones of inhibition when combined with POS or VRC, but not with FLC.

The *C. auris* clinical isolates tested did not give zones of inhibition when CLO or its analogs were added on a disk at 50 nmol/disk. CBS10913^T^ is susceptible to azoles (Table 2), with FLC giving the highest MIC (2 mg/L). The combination of CLO and its analogs (50 nmol/disk) with POS, VRC, or FLC did not enlarge the inhibition zone around these disks. AR0389 carries the Erg11 amino acid substitution Y132F and overexpresses CauCdr1, with these phenotypes elevating azole MIC values collectively [39,87,88,89]. CLO in combination with POS, VRC, or FLC did not increase inhibition zone size. In contrast, M19, M20, or *N*-(3-(2,4-dichlorophenoxy)propyl)-*N*-methylprop-2-en-1-amine (M25) combined with POS gave larger zones of inhibition, but for VRC only, M19 increased inhibition zone size. None of the CLO analogs in combination with FLC enlarged the zone of inhibition (Table S1).

### 3.3. Antifungal Activity of Modulators on Recombinant Strains Expressing Efflux Pumps

The antifungal activity of known and potential modulators of drug efflux were evaluated quantitatively by determining the MIC values they elicited with recombinant strains. The host strain ADΔΔ and the strain overexpressing CauErg11 were used as controls that should be unaffected by modulators of drug efflux. As expected, the Cdr1 inhibitors ENB and BEA plus CLO and M19 resulted in MICs comparable to the control strains (Table 3). The Cdr1-overexpressing strains CauCdr1 and CaCdr1B required several-fold higher concentrations of PON, OME, and RAB than the control strains to achieve sufficient inhibition, indicating that these compounds are substrates of CauCdr1 and CaCdr1B. OME also appeared to be a substrate of CauMdr1 and CaMdr1A. M25 appeared to be a weak substrate of CaMdr1A and CaCdr1B and an even weaker substrate of CauMdr1 and CauCdr1. Neither CLO nor M19 appear to be substrate of these efflux pumps.

### 3.4. Effect of Efflux Pump Inhibitors on Recombinant Strains

**Figure 1:**
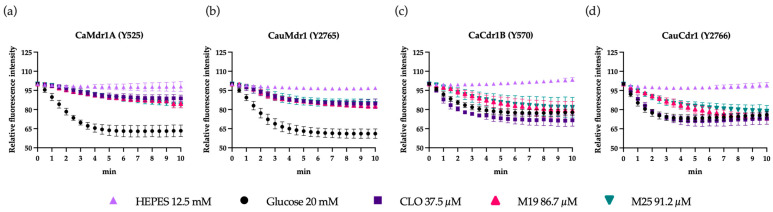
Kinetics of Nile Red efflux by recombinant *S. cerevisiae* strains overexpressing Mdr1 and Cdr1. (**a**,**b**) CLO, M19, and M25 inhibit the rate and steady state level of Nile Red efflux in Mdr1-overexpressing strains. (**c**,**d**) M19 and M25 maximally inhibit Cdr1 efflux after 2.5 min but fail to modify the steady state level of efflux at 10 min. CLO does not significantly modify drug efflux in these strains. Shown is the average of three biological replicates (*n* = 3) in technical duplicates with standard deviation.

The glucose-dependent efflux activity of Cdr1 and Mdr1 in recombinant *S. cerevisiae* cells was monitored by measuring the fluorescence intensity of the efflux pump substrate Nile Red (Figure 1). Nile Red is more fluorescent when bound to the cell membrane than in aqueous solution [90]. Efflux pump inhibitors prevent cells from dynamic clearing of Nile Red and, therefore, reaching and/or maintaining a lower steady state level of fluorescence.

**Figure 2:**
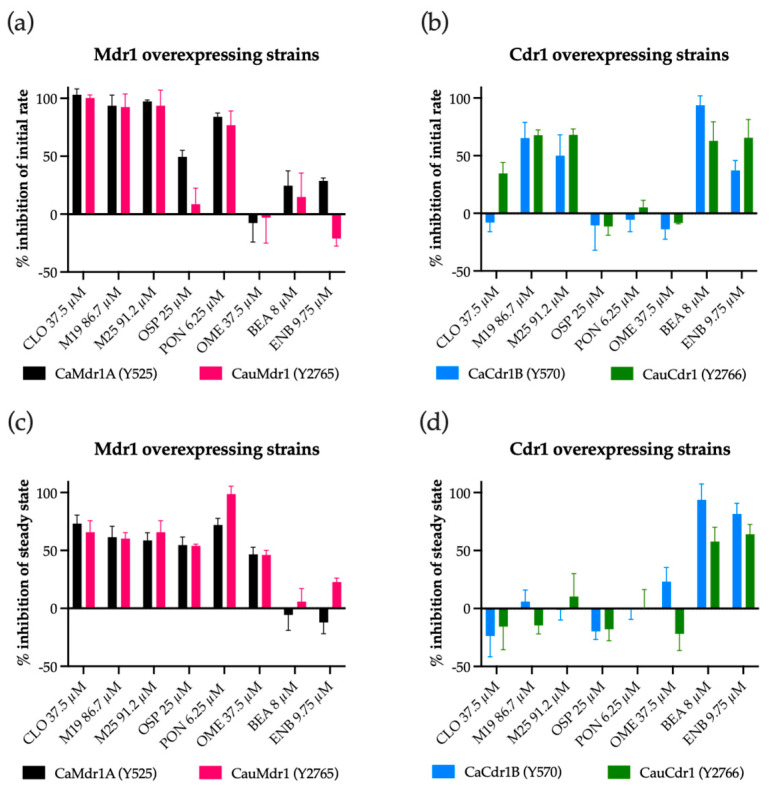
Nile Red efflux in recombinant strains overexpressing Mdr1 and Cdr1 showing inhibition of the (**a**,**b**) initial rate within the first minute and (**c**,**d**) steady state at 10 min compared to untreated glucose control. Results of three biological replicates (*n* = 3) in technical duplicates are shown. Error bar represents mean with standard deviation.

Nile Red-loaded cells were pre-incubated in serial dilutions of modulators in HEPES buffer and dye efflux initiated by adding 20 mM glucose as energy source. Figure 1 and Figure S2 show the kinetics of Nile Red efflux and Figure 2 the initial rates and steady states obtained after 10 min for strains overexpressing Mdr1 or Cdr1, while efflux in control strain ADΔΔ was not detectable (Figure S3).

For the recombinant strains overexpressing Mdr1, all compounds tested, except for the Cdr1-specific inhibitors BEA and ENB and the proton pump inhibitor OME (Figure S2a,b), immediately reduced the rate of Nile Red efflux (Figure 2a,) and reduced the steady state level of efflux after 10 min by ≥50% (Figure 2c). Acid-activated OME was slower in action, showing efflux rate inhibition after 3 min followed by a substantial steady state modification (Figure S2a,b). BEA and ENB did not affect either the rate of efflux or the final steady state.

The CLO analogs M19 and M25 reduced the efflux activity in the strain overexpressing CaCdr1 by >45% during the first 2.5 min (Figure 1c) and reached a stable steady state after 8 min comparable to the control (Figure 2d and Figure S2c). No other compounds, apart from BEA and ENB, affected either the rate efflux or the steady state efflux activity of CaCdr1. Both M19 and M25 inhibited the initial rate of Nile Red efflux of CauCdr1 by >60% (Figure 2b), but this inhibition did not reduce the steady state of Nile Red efflux after 10 min (Figure 2d). Only BEA and ENB strongly reduced the steady state level of Nile Red efflux after 10 min in the strain overexpressing CauCdr1.

### 3.5. Effect of the Modulators CLO, M19, and M25 on Cdr1 ATPase Activity In Vitro

**Figure 3:**
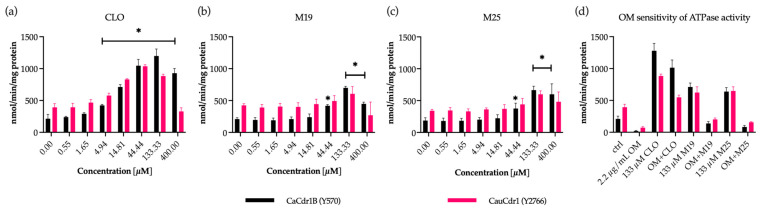
ATPase activity of crude membrane preparations from recombinant strains overexpressing Cdr1 with and without inhibitors (**a**) CLO, (**b**) M19, (**c**) M25. The ATPase activity sensitive to OM (2.2 µg/mL) (**d**) was determined in absence and presence of the indicated concentrations of CLO, M19, or M25. The experiment was carried out in technical duplicates in three biological replicates (*n* = 3). Bar represents mean value with standard deviation. Statistical analysis was performed using GraphPad Prism 9 with Two-Way ANOVA Dunnett’s multiple comparison test. (*) significant *p* value < 0.05 of treated crude membranes compared to untreated control.

The dose-responses to test compounds on CaCdr1 and CauCdr1 ATPase activity in crude membrane preparations from recombinant yeast strains are presented in Figure 3a–c. CLO (≥4.94 µM), M19 (≥44.4 µM), and M25 (≥44.4 µM) significantly enhanced the ATPase activity in membranes containing overexpressed CaCdr1. CLO (≥4.94–133 µM), M19 (133 µM), and M25 (133 µM) increased the ATPase activity in membrane containing overexpressed CauCdr1.

Previous studies have shown that OM inhibits the ATPase activity of CaCdr1 and CauCdr1 [25,70]. Therefore, OM was used to show that ATPase activation caused by 133 µM CLO, M19, and M25 was specific (Figure 3d). The ATPase activity maximally activated by M19 or M25 was almost completely inhibited by OM for both membrane preparations from Cdr1-overexpressing strains. The ATPase activities of CauCdr1 and CaCdr1 were activated 2-fold more by CLO than by M19 and M25, but the CLO activated activity was only 50% inhibited by OM for CauCdr1 and 25% for CaCdr1. Thus, the OM-sensitive activities activated by CLO and its two analogs were comparable.

### 3.6. Combinatorial Effect of Azoles and Modulators on Recombinant Strains Overexpressing Mdr1 and Cdr1

The recombinant *S. cerevisiae* strains Y2765 and Y2766 were used determine the effect of modulators in combination with azoles (POS, VRC, ISA) on the *C. auris* efflux pumps CauMdr1 and CauCdr1 (Table S2). Recombinant *S. cerevisiae* strains expressing CaMdr1A (Y525) and CaCdr1B (Y570) were also tested to see if the phenotypic responses observed with the *C. auris* efflux pumps are comparable with those of *C. albicans* (Table S3). For these experiments, the CauErg11-overexpressing strain Y2767 was used as a negative control (Table S4). The potential modulators tested were CLO, M19, M25, OME, RAB, PON, and OSP. The established Cdr1 inhibitors BEA and ENB were used as positive controls for efflux activity mediated by CaCdr1 and CauCdr1.

The FICI values obtained for the recombinant strains overexpressing CauCdr1 or CauMdr1 are detailed in Table S2 and summarized in Table 4. Synergy between compounds is defined as FICI ≤ 0.5 (see Materials and Methods). While none of the compounds tested were antagonists, CLO, M19, M25, and OME each significantly increased susceptibility (>3-fold) of the CauMdr1-expressing strain to POS, VRC, and ISA. Only OME and M25 synergized the activity of POS, VRC, and ISA (Table 4), with the VRC/M25 combination exhibiting weakest synergy (FICI = 0.50). The impact of OME on drug susceptibility was expected as inhibition of the fungal plasma membrane proton pump Pma1 should limit the plasma membrane electrochemical gradients and, hence, the activity of Mdr1. The VRC/CLO, ISA/CLO, VRC/PON, and POS/RAB combinations also gave FICI values ≤ 0.5. All other combinations showed no interaction (Table 4). The *C. albicans* Mdr1-overexpressing strain Y525 showed similar results, with M25 synergizing the activity of the three antifungals tested. Furthermore, synergy was obtained with the VRC/CLO, ISA/CLO, and VRC/PON combinations but not POS/RAB (Table S3). In addition, M19 synergized the activity of ISA. In contrast with CauMdr1, OME failed to synergize the activity of the three antifungals in the strain expressing CaMdr1. As expected, the Cdr1-specific inhibitors BEA and ENB gave “no interaction” scores with both Mdr1-overexpressing strains. However, POS significantly increased (2-fold) susceptibility to both ENB and BEA, while VRC increased susceptibility to BEA due to CauMdr1 expression (Table S2) and to both ENB and BEA due to CaMdr1 expression (Table S3). As ENB and BEA are not substrates of Mdr1, these increased susceptibilities must be due to an alternative mechanism.

CLO, M19, M25, and OME each synergized the activity of POS when tested with the strain overexpressing CauCdr1. The combinations VRC/M19 and VRC/OME also gave synergistic effects. BEA and ENB gave strong synergy as expected of these Cdr1 inhibitors (Table 4).

The recombinant strains overexpressing *C. albicans* Cdr1 and CauCdr1 gave equivalent synergy results for modulator combinations with POS, except OME (FICI = 0.51). While M19 and OME gave synergy with VRC for the strain expressing CauCdr1, CLO and OME conferred synergy with VRC for the strain expressing CaCdr1. The ISA/OME combination gave synergy for the strain expressing CaCdr1 but not CauCdr1 (Tables S2 and S3). The strain Y2767 overexpressing CauErg11 as a negative control gave “no interaction” FICI values for all combinations tested (Table S4).

### 3.7. Combinatorial Effect of Azoles and CLO, M19, and M25 on C. auris Clinical Isolates

CLO, M19, or M25 in combination with azoles were tested against the *C. auris* wild-type clade II clinical isolate CBS10913^T^, and the clade I clinical isolates CBS12875, and AR0389. Azole/CLO combinations gave “no interaction” (Table 5) with both clade I strains, except POS/CLO with AR0389 which gave borderline synergy (FICI = 0.50). However, all azole/M19 or azole/M25 combinations gave strong synergy for each clade I strain (FICI ≤ 0.33, Table S5). CBS10913^T^ is the susceptible wild-type strain not carrying the Erg11 amino acid substitution Y132F. Nevertheless, CBS10913^T^ showed synergy with M19 in combination with all azoles, except VRC, and M25 synergized with POS and VRC (Table 5).

The echinocandin MFG is not subject to drug efflux in yeast [91] and, therefore, served as negative control. All MFG/modulator combinations gave “no interaction” for the three strains tested.

### 3.8. Effects on C. albicans Clinical Isolates of Azoles in Combination with M19 or M25

The response to azoles in combination with M19 and M25 of the related *C. albicans* clinical isolates TL1 and TL3 [71] and laboratory strain SGY-243 [72] and the derivative strain FR2 [28] were determined. TL1 and SGY-243 are susceptible to FLC, while TL3 and FR2 are resistant to FLC due to increased expression of *CDR1* or *MDR1*, respectively. Checkerboard assays were carried out with the antifungals POS and VRC in combination with M19 or M25. None of these combinations gave synergy (Table 6). Although antifungal susceptibilities were not increased by the combinations, susceptibility to M19 and M25 increased at least 100-fold, with the exception that for *C. albicans* TL3 the susceptibility to M25 increased 2.5-fold in combination with VRC (Table S6).

## 4. Discussion

### 4.1. Expression of Azole Resistance Determinants in C. auris Clade I and Clade II Clinical Isolates

Understanding of the mechanistic basis of drug resistance in clinical isolates in *C. auris* is needed for the development of new therapies. Recombinant *S. cerevisiae* strains functionally expressing individual enzymes that contribute to azole resistance in *C. auris* provide insight into the mechanisms determining azole susceptibility patterns of clinical isolates from all clades. A similar reductionist approach may enable reconstruction of cross- and pan-resistance patterns that are rarer than azole resistance.

In the present study, *C. auris* clade II strain CBS10913^T^ has been considered as a wild-type reference strain that does not overexpress CauErg11, CauCdr1, or CauMdr1 and is, therefore, susceptible to all azole antifungals. Strains AR0389 and CBS12875 belong to clade I and both carry the Erg11 Y132F amino acid substitution that confers significant azole resistance to the short-tailed azoles FLC and VRC but not the long-tailed azoles ITC and POS. The CauErg11 Y132F mutation has also been found in clades IV and V [3,19], while the CauErg11 K143R mutation can occur in clades I and IV [3,44]. Both mutations show resistance to the short-tailed azoles and susceptibility to the long-tailed azoles [25]. Strain AR0389 has been shown to overexpress CauCdr1 [40], but the overexpression of CauErg11 or the efflux pumps CauMdr1 and CauCdr1 has yet to be determined for strain CBS12875. Our comparison of the drug susceptibility phenotypes of the clade I and clade II clinical isolates with the recombinant strains [25] show resistance pattern similarities between AR0389 and CBS12875 for CauCdr1 but not CauErg11 or CauMdr1 overexpressed in *S. cerevisiae*. The recombinant *S. cerevisiae* strains with azole resistance mediated by overexpressed CauErg11 Y132F or CauMdr1 show reduced VRC susceptibility compared to the host strain (25- and 129-fold, respectively) but do not show reduced susceptibility to ITC or POS. In contrast, overexpression of CauCdr1 gave even higher-level resistance compared to the host strain for VRC (258-fold) and significant resistance to both ITC (>47-fold) and POS (5-fold) (Table 2). Compared to the clade II clinical isolate CBS10913^T^, strains AR0389 and CBS12875 showed much higher MIC_80_ values for FLC and VRC and more modest but significantly higher MIC_80_ values for ITC and POS (Table 2 and confirmed in Table S5). These data indicate that the expression of CauCdr1 dominates drug resistance in the clade I clinical isolates and that more functional CauCdr1 is expressed in AR0389 than in CBS12875. CauErg11 Y132F expression can also be expected to make a minor contribution to FLC and VRC resistance but not ITC and POS resistance, while an additional but minor contribution to FLC and VRC resistance by CauMdr1 cannot be excluded.

### 4.2. Identification and Characterization of CLO Analogs as Candidate Modulators of Drug Efflux in Combination Therapy

Combination therapy has been used successfully to treat diseases, such as HIV [92], malaria [93], and tuberculosis [94], where monotherapy had resulted in the rapid emergence of drug resistance. While the use of combination therapy has been posited as a strategy to increase the efficacy and the lifespan of existing antifungal drugs [95], combination therapies have yet to be widely applied to combat antifungal resistance, e.g., cryptococcal infections and *Candida* endocarditis [96,97].

The out-of-patent monoamine oxidase A (MAO-A) inhibitor CLO, which is related to the antidepressant drug Pargyline, was shown to synergize with azole substrates of the *C. glabrata* and *C. albicans* Cdr1 and Mdr1 efflux pumps [51]. The present study has shown CLO synergized with the azoles POS and VRC in recombinant *S. cerevisiae* strains overexpressing Ca/CauMdr1A and Ca/CauCdr1B but failed to synergize these drugs with *C. auris* clinical isolates (except for the borderline synergy FICI = 0.50 in combination with POS for strain AR0389). Among six structural analogs of CLO (Figure S1), a screen using an agarose diffusion assay identified compounds M19 and M25 as candidate modulators of azole inhibition of the growth of *C. auris* clinical isolates (Table S1.) Unlike CLO, both M19 and M25 gave synergy with the azoles in checkerboard assays for the *C. auris* clade I clinical isolates tested. Inhibition of Nile Red efflux mediated by CauMdr1 and CauCdr1 in the recombinant *S. cerevisiae* strains and in checkerboard assays with the recombinant strains showed that M25 synergized with azole substrates of both the CauCdr1 and CauMdr1 efflux pumps. Despite inhibiting the rate of glucose-dependent Nile Red efflux mediated by CauCdr1 and CauMdr1, M19 synergized with azole substrates effluxed by CauCdr1 but not CauMdr1 in the checkerboard assay. As expected from its ability to reduce efflux in the recombinant *S. cerevisiae* strains expressing either CauCdr1 or CauMdr1, M25 synergistically reduced azole resistance in the clade I clinical isolates. M19 was slightly more effective than M25 in synergizing with the azole drugs tested in the clade I clinical isolates, despite not synergizing efflux in the recombinant *S. cerevisiae* strains overexpressing Mdr1. These results are consistent with CauCdr1 dominating drug efflux in the clade I strains.

M19 and M25 appear to be weak substrates of the CauMdr1 and CauCdr1 drug efflux pumps, but their inhibitory mode of action is not fully understood. They significantly inhibit the rate of drug efflux by Mdr1 or Cdr1 (monitored by Nile Red efflux) and reduce steady-state efflux by both CaMdr1 and CauMdr1, but they do not alter the steady state levels of efflux obtained by CaCdr1 and CauCdr1. CLO, M19, and M25 stimulate comparably the OM-sensitive ATPase activity of Cdr1. M19 and M25 activated the OM-sensitive activity of CaCdr1 ~100% and CauCdr1 ~50%. This suggests that M19 and M25 uncouple Cdr1 ATPase activity from the active transport of substrates. This limits the rate of Nile Red or azole efflux but is insufficient to substantially affect the steady state of substrate efflux for Nile Red measured at 10 min. We note that a significantly higher proportion of CaCdr1 activation by CLO is not inhibited by OM and may involve other unknown factors. Furthermore, in contrast to the ~50% initial rate inhibition of CauCdr1-mediated Nile Red efflux by M19 and M25 at ~90 µM, CLO at both 37.5 µM and 75 µM inhibited the initial rate of Nile Red efflux by <30% for CauCdr1 and slightly activated the initial rate for CaCdr1 (Figure S4).

The lead structure CLO is known as a mechanism-based inhibitor that binds covalently to the N5 of the cofactor FAD in the active site of its original target protein MAO-A due to the chemical reactivity of its propargylamine entity [98]. Some of the CLO analogs found to be inactive in our screen bear functional groups which should also allow covalent binding to nucleophilic centers (NH, SH, and OH moieties) in target proteins (nitrile in M18 and epoxide in M20). The active analogs, however, bear allylamine (M19) and cyclopropylmethylamine (M25) residues in place of CLO’s propargylamine (Figure S1). Some publications have indicated that allylamines can bind covalently to target molecules as well [99,100]. While cyclopropylamines have been reported to bind covalently to proteins, the homologous cyclopropylmethylamines (see M19) do not show this reactivity [101]. Therefore, in the context of the present study, the binding sites of CLO, M19, and M25 in their target proteins and their molecular modes of binding (reversible or irreversible) are not obvious and deserve further investigation.

Unlike mammalian ABC transporters, the ATPase activity of yeast ABC transporters, such as Cdr1, is not stimulated by the drugs they transport [102,103,104]. Azole drugs bind to Cdr1 in the absence of ATP hydrolysis, with ATP hydrolysis required to complete the reaction cycle [105]. As M19 and M25 are weak substrates of Cdr1, their activation of the OM-sensitive ATPase activity of Cdr1 is likely to involve interaction with the enzyme outside the drug binding sites, e.g., together with the adjacent lipid bilayer or in the nucleotide-binding domains and, thereby, modifying the conformation of the Cdr1 to favor uncoupled, substrate-independent cycles of ATP hydrolysis.

The identification and characterization of the CLO analogs M19 and M25 as inhibitors of drug efflux and as candidates for further development has significant limitations. First, as a small number of clinical isolates were tested and the confirmation of the effects of M19 and M25 will require tests with greater numbers of clinical isolates, i.e., not only from clades I and II but also from clades III–V. For example, we expect M19 and M25 to synergize with azole drugs in strains from clades I and IV due to the dominance of Cdr1-mediated azole efflux and for M25 to affect Mdr1-dependent azole efflux detected in clade III. Furthermore, M19 and M25 appear specific for *C. auris* drug efflux and do not affect drug efflux in the *C. albicans* clinical isolates tested, despite showing in vitro activity against CaCdr1. Second, the concentrations of M19 and M25 needed to achieve synergy with azole drugs may exceed relevant clinically achievable doses. Studies in the *Galleria mellonella* infection model could shed light on this matter and provide preliminary test of the feasibility of using these new compounds in animals. Third, M19 and M25 do not behave identically despite considerable structural similarity. The modes of action of M19 and M25 are not known and may not be identical, but their uncoupling of Cdr1 ATPase activity by affecting the fluidity of the plasma membrane adjacent to CauCdr1 is an intriguing possibility. This can be investigated using fluorescence polarization measurements with suitable probes of plasma of membrane lipid fluidity. Fourth, the checkerboard method of susceptibility determinations has important drawbacks [106]. Incomplete guidelines for *C. auris* MIC endpoint determinations [86] and checkerboard assays based on serial double dilutions can lead to differences in FICI values that affect interpretation, e.g., at the boundary between “synergy” and “no interaction”. Finally, the ADMET properties of M19 and M25 would need to be carefully evaluated during drug development as CLO has known interactions with key liver drug metabolizing cytochrome P450 enzymes and is a substrate of P-glycoprotein.

### 4.3. Characterization of Other Potential Modulators of Drug Efflux

Inspired by previous studies with *C. albicans* and *C. auris* clinical isolates, PON, OME, RAB, and OSP were tested as modulators of drug efflux on recombinant *S. cerevisiae* strains overexpressing Mdr1 and Cdr1 from *C. albicans* and *C. auris*.

Liu et al. [49] used the tyrosine kinase inhibitor and anti-cancer drug PON with FLC against the recombinant *S. cerevisiae* strains and clinical isolates of *C. albicans* and *C. neoformans*. The synergies detected with the *S. cerevisiae* strains indicate PON is effluxed competitively by the Pdr5 drug transporter and are consistent with the previously observed inhibition of the expression of the plasma membrane proton pump Pma1 [49]. For example, PON acts synergistically with VRC in *S. cerevisiae* strains expressing Mdr1 but not Cdr1 during glucose-dependent Nile Red efflux (Figure 2d) and in checkerboard assays (Table 4 and Table S3). The significantly (>6-fold) higher MIC_80_ for PON in our *S. cerevisiae* strains expressing either CauCdr1 or CaCdr1 than the host strain or strains expressing CaMdr1 and CauMdr1 indicate PON is a substrate of these ABC transporter homologs of Pdr5. Our results are consistent with PON rapidly inhibiting VRC transport via Mdr1, with the specificity in checkboard assays likely due to longer-term reduction in membrane potential caused by reduced Pma1 levels. Further research will be required to determine whether FLC susceptibility is affected by direct competition with PON efflux mediated by CauCdr1 or CauMdr1.

The selective estrogen receptor modulator OSP was previously shown to act synergistically with ITC (but not FLC or VRC) against *C. auris* clinical isolates from clade I and IV [52]. It was suggested that OSP might directly interfere with efflux pumps of *C. auris*. We found OSP inhibits Nile Red efflux in *S. cerevisiae* strains overexpressing CaMdr1 or CauMdr1 but not strains overexpressing CaCdr1 or CauCdr1 (Figure 2c,d). However, checkerboard analysis gave “no interaction” FICI values for azoles and OSP with all the *S. cerevisiae* strains overexpressing Mdr1 and Cdr1 (Table 4 and Table S3). As ITC is a substrate of Cdr1 and a poor substrate of Mdr1, the activity of OSP is unlikely to involve a direct effect on CauCdr1-mediated drug efflux.

Proton pump inhibitors (PPIs), such as OME and RAB, have antifungal activity against yeast [50,75,107]. We found synergy between OME and azole drugs for all efflux pumps tested at pH 3.5 except CaMdr1. As OME targets Pma1 [75], inhibition of this enzyme depletes plasma membrane electrochemical gradients (proton gradient and membrane potential) and, thus, diminishes the activity of Mdr1. A longer-term reduction in nutrient uptake may also reduce metabolic activity and the levels of ATP available for Cdr1-mediated drug efflux. The reason why OME did not target CaMdr1 has yet to be elucidated, but this may be due to the low pH used to activate the prodrug and keep its sulfenamide product stable. A second PPI, RAB, showed good efficacy against the CauMdr1-overexpressing strain (Table 4), and this finding was confirmed in a glucose-dependent Nile Red efflux assays (Figure 2c). However, RAB did not block CauCdr1-mediated efflux (Figure 2d) or synergize with other azoles except POS efflux by CauMdr1 (Table 4). An interesting dose-dependent effect of RAB was observed by Liu et al. [76] and confirmed by Lu et al. [50]. They found high concentrations of RAB gave weaker growth inhibition than lower concentrations. It is possible that lower concentrations of RAB than we tested will inhibit azole efflux in the CauCdr1-overexpressing strain Y2766.

The MICs of the potential CauCdr1 substrates PON, OME, and RAB (their individual MIC was several times higher for the CauCdr1-expressing strain than the host strain, Table 3) were substantially reduced (e.g., 400-fold for OME) when combined with POS or ISA (Table S2). The same effect was observed in combination with VRC, except for OME, where the MIC was reduced by 4-fold (Table S2). Comparable observations made for CaCdr1 and azole/OME combinations (Table S3) suggest these compounds compete in the Cdr1 substrate-binding site with azole drugs having higher affinity than OME.

It was also found that low pH conditions used to test the effect of OME drastically reduced the MICs of Cdr1-expressing strains to POS and ISA, but not VRC. This supports the hypothesis that OME competes with azoles at the drug-binding site of Cdr1 and that POS and ISA have greater affinity for the protein under these conditions.

## 5. Conclusions

Our experimental approach has identified the multi-targeting Clorgyline analogs M19 and M25 which synergize with azole drugs to increase their efficacy against efflux-based resistance in two *C. auris* clade I strains.

Collectively, the present study provides a starting point to develop a combination therapy that combats azole resistance in *C. auris* mediated by Erg11 and Cdr1. By targeting both Erg11 with long-tailed azoles, such as POS (which is not affected by either the CauErg11 Y132F or K143R mutations), and inhibiting antifungal efflux with M19 or M25, the experimental therapy has the advantage of delivering higher intracellular POS concentrations than standard azole monotherapy and, thereby, further reducing opportunity for the development of azole resistance.

To our knowledge, this is the first study, with mechanistic investigations using recombinant *S. cerevisiae* strains and tests in clinical isolates, that combines *C. auris* specific efflux pump inhibitors with azole drugs that are least susceptible to the common mutations in CauErg11. Further research is needed to determine the mode of action of the CauMdr1 inhibitors and CauCdr1 uncouplers M19 and M25 and their effects on all five clades of *C. auris*.

## Figures and Tables

**Table 1:** Strains used in this study.

Strain	Genotype	Description	Reference/Source
Y1857 (ADΔΔ)	*MAT*α *pdr1-3 his1 Δyor1::hisG Δsnq2::hisG Δpdr10::hisG Δpdr11::hisG Δycf1::hisG Δpdr3::hisG Δpdr5::hisG Δpdr15::hisG, Δura3 Δhis1::dpl2000*	*S. cerevisiae* host	[69]
Y525 (CaMdr1A)	AD *Δpdr5::CaMDR1A*	*C. albicans* Mdr1A overexpressed	[70]
Y570 (CaCdr1B)	AD *Δpdr5::CaCDR1B*	*C. albicans* Cdr1B overexpressed	[70]
Y2765 (CauMdr1)	ADΔΔ *Δpdr5::CauMDR1*	*C. auris* Mdr1 overexpressed	[25]
Y2766 (CauCdr1)	ADΔΔ *Δpdr5::CauCDR1*	*C. auris* Cdr1 overexpressed	[25]
Y2767 (CauErg11)	ADΔΔ *Δpdr5::CauERG11*	*C. auris* Erg11 overexpressed	[25]
CBS10913^T^	Wild type, clade II	*C. auris* clinical isolate	Westerdijk Fungal Biodiversity Institute
CBS12875	Erg11 Y132F, clade I	*C. auris* clinical isolate	Westerdijk Fungal Biodiversity Institute
AR0389	Erg11 Y132F, *CDR1* overexpression, clade I	*C. auris* clinical isolate	Antimicrobial Resistance Isolate Bank
TL1	FLC susceptible	*C. albicans* clinical isolate	[71]
TL3	FLC resistant	*C. albicans* clinical isolate	[71]
SGY-243	*ade2/ade2 Δ* *ura3::ADE21/Δ* *ura3::ADE2*	Mutagenized *C. albicans* isolate	[72]
FR2	SGY-243 passaged in FLC	*C. albicans* Mdr1 overexpressed	[28]

**Table 2:** MIC values of *C. auris* clinical isolates used in this study.

Strain	FLC	VRC	ITC	POS	ANA	MFG	AMB
CBS10913^T^	2.0 (S)	0.008 (n.d.)	0.016 (n.d.)	0.03 (n.d.)	0.03 (S)	0.03 (S)	0.5 (S)
CBS12875	>64.0 (R)	2.0 (n.d.)	0.25 (n.d.)	0.06 (n.d.)	0.125 (S)	0.125 (S)	4.0 (R)
AR0389	>64.0 (R)	4.0 (n.d.)	0.5 (n.d.)	0.25 (n.d.)	0.25 (S)	0.125 (S)	1.0 (S)

MIC values shown in mg/L are the mean of three biological replicates (*n* = 3). S susceptible, R resistant, n.d. not defined.

**Table 3:** MIC values for recombinant *S. cerevisiae* strains treated with modulators.

Drug Class	Compound	CauErg11	CauMdr1	CauCdr1	CaMdr1A	CaCdr1B	ADΔΔ
Cdr1 inhibitor	Enniatin B (ENB)	15.8	15.5	10.7	16.8	5.4	14.4
	Beauvericin (BEA)	11.0	9.0	6.7	11.8	11.8	11.2
Tyrosine kinase inhibitor	Ponatinib (PON)	8.3	7.3	50	8.3	>50	5.2
PPI	Omeprazole (OME)	58.3	315	1900	500	>6000	75
	Rabeprazole (RAB)	278	278	>1875	156	>1875	29
Estrogen receptor modulator	Ospemifene (OSP)	>250	>250	>250	>250	>250	>250
MAO-A inhibitor	Clorgyline (CLO)	150	99	130	99	140	137
Clorgyline analogs	M19	86.7	86.7	98.3	86.7	86.7	86.7
	M25	137	182	228	273	273	137

Average of three replicates (*n* = 3) in µM. > MIC higher than value shown.

**Table 4:** FICI summary for recombinant strains overexpressing CauMdr1 or CauCdr1 with azole drugs in combination with modulators.

	Recombinant *S. cerevisiae*	
Reagents	CauMdr1 Y2765	CauCdr1 Y2766
POS/CLO	NI	SYN
POS/M19	NI	SYN
POS/M25	SYN	SYN
POS/PON	NI	NI
POS/OSP	NI	NI
POS/OME	SYN	SYN
POS/RAB	SYN	NI
POS/ENB	NI	SYN
POS/BEA	NI	SYN
VRC/CLO	SYN	NI
VRC/M19	NI	SYN
VRC/M25	SYN	NI
VRC/PON	SYN	NI
VRC/OSP	NI	NI
VRC/OME	SYN	SYN
VRC/RAB	NI	NI
VRC/ENB	NI	SYN
VRC/BEA	NI	SYN
ISA/CLO	SYN	NI
ISA/M19	NI	NI
ISA/M25	SYN	NI
ISA/OSP	NI	NI
ISA/OME	SYN	NI

Detailed information on FIC and FICI determinations is presented in Table S2. Synergy, SYN; No interaction, NI.

**Table 5:** FICI summary for *C. auris* clinical isolates with azole drugs in combination with CLO, M19, and M25.

	*C. auris*		
Reagents	CBS12875	AR0389	CBS10913^T^
POS/CLO	NI	SYN	NI
POS/M19	SYN	SYN	SYN
POS/M25	SYN	SYN	SYN
VRC/CLO	NI	NI	NI
VRC/M19	SYN	SYN	NI
VRC/M25	SYN	SYN	SYN
ITC/CLO	NI	NI	NI
ITC/M19	SYN	SYN	SYN
VT-1161/CLO	NI	NI	NI
VT-1161/M19	SYN	SYN	SYN
MFG/CLO	NI	NI	NI
MFG/M19	NI	NI	NI

Detailed information on FIC and FICI determinations is presented in Table S5.

**Table 6:** FICI summary for *C. albicans* clinical isolates with azole drugs in combination with M19 and M25.

	*C. albicans*			
Reagents	TL1	TL3	SGY243	FR2
POS/M19	NI	NI	NI	NI
POS/M25	NI	NI	NI	NI
VRC/M19	NI	NI	NI	NI
VRC/M25	NI	NI	NI	NI

Detailed information on FIC and FICI determinations are presented in Table S6.

## Data Availability

Not applicable.

## Supplementary Materials

The following supporting information can be downloaded at: https://www.mdpi.com/article/10.3390/jof9060663/s1. Figure S1: Chemical structures of Clorgyline and its analogs; Supplement 1: Synthesis of the Clorgyline analogs; Table S1: Zone of inhibition ratio in disk diffusion screen; Figure S2: Effects of efflux modulators on glucose-dependent Nile Red efflux by recombinant strains; Figure S3: Glucose-dependent Nile Red efflux by the control strain ADΔΔ; Figure S4: Effect of 75 µM CLO on glucose-dependent Nile Red efflux by recombinant strains; Table S2: Checkerboard susceptibility assays of recombinant strains overexpressing CauCdr1 and CauMdr1 for azole drugs in combination with modulators; Table S3: Checkerboard susceptibility assays of recombinant strains overexpressing CaCdr1 and CaMdr1 for azole drugs in combination with modulators; Table S4: Checkerboard susceptibility assays for recombinant strains overexpressing CauErg11 for azole drugs in combination with modulators; Table S5 Checkerboard susceptibilities assays for *C. auris* clinical isolates for azole drugs in combination with CLO, M19, and M25; Table S6: Checkerboard susceptibilities assays for *C. albicans* clinical isolates for azole drugs in combination with M19 and M25.
