# Supplementary material for: Clorgyline Analogs Synergize with Azoles against Drug Efflux in Candida auris
Source: J Fungi (Basel). 2023 Jun 13;9(6):663. doi: 10.3390/jof9060663 (PMC10303049; doi:10.3390/jof9060663)
Supplement: Supplementary file 1 [file jof-09-00663-s001.zip › jof-2426731-supplementary.pdf]

Journal of *Fungi*

MDPI

Supplemental Materials

Clorgyline Analogs Synergize with Azoles Against Drug Efflux in *Candida auris*

Stephanie Toepfer [1], Michaela Lackner [2], Mikhail V. Keniya [1,3], Lisa-Maria Zenz [2], Marianne Friemert [4], Franz Bracher [4], and Brian C. Monk [1,*]

[1] Sir John Walsh Research Institute, Faculty of Dentistry, University of Otago, Dunedin 9016, New Zealand; stephanie@toepfer.co, stephanie.toepfer@postgrad.otago.ac.nz
[2] Medical University of Innsbruck, Institute of Hygiene and Medical Microbiology,6020 Innsbruck, Austria; michaela.lackner@i-med.ac.at
[3] Hackensack Meridian Health Center for Discovery and Innovation, Nutley, NJ 07110, USA; mikhail.keniya@hmh-cdi.org
[4] Department of Pharmacy, Center for Drug Research, Ludwig-Maximilian University of Munich, 81377 Munich, Germany; franz.bracher@cup.lmu.de
* Correspondence: brian.monk@otago.ac.nz

check for updates

Citation: Toepfer, S.; Lackner, M.; Keniya, M.V.; Zenz, L.-M.; Friemert, M.; Bracher, F.; Monk, B.C. Clorgyline Analogs Synergize with Azoles against Drug Efflux in *Candida auris*. *J. Fungi* **2023**, *9*, 663. https://doi.org/10.3390/jof9060663

Academic Editors: Yanan Zhao, Karen Joy Shaw and James M. Balkovec

Received: 16 May 2023
Revised: 8 June 2023
Accepted: 9 June 2023
Published: 13 June 2023

Copyright: © 2023 by the authors. Licensee MDPI, Basel, Switzerland. This article is an open access article distributed under the terms and conditions of the Creative Commons Attribution (CC BY) license (https://creativecommons.org/licenses/by/4.0/).

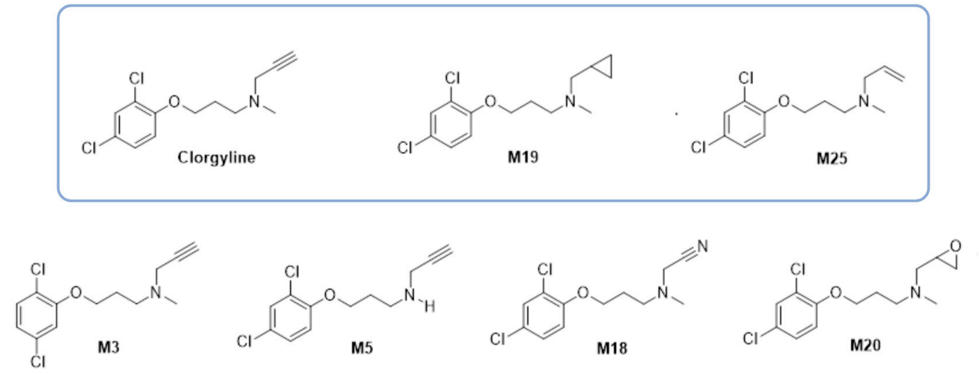

Figure S1. Chemical structures of Clorgyline and its analogs.

Supplements 1: Synthesis of the Clorgyline analogs

The *N*-nor analog **M5** was synthesized from intermediate **1** and propargylamine following a published protocol [1]. The preparation of secondary amine **2** by reaction of bromo compound **1** with methylamine gave acceptable yields only if catalytic amounts of KI were added.

Starting from intermediate **2**, four Clorgyline analogs were prepared in which the propargyl group is replaced by other functional groups of comparable size: reaction with chloroacetonitrile gave cyanomethyl analog **M18**, (chloromethyl)cyclopropane yielded compound **M19**, epichlorohydrine gave epoxide **M20**. The yield of **M20** was rather poor due to a strong tendency of this epoxy compound to undergo further nucleophilic attack by a second equivalent of the secondary amine **2**. *N*-Alkylation of **2** with allyl chloride gave *N*-allyl analog **M25**. It is noted that attempts to prepare **M25** using allyl bromide failed due to extensive overalkylation giving a quaternary ammonium salt.

The analog **M3**, having a distinct chlorination pattern on the aromatic ring, was prepared from the known bromoether **3** [2] by reaction with *N*-methylpropargylamine.

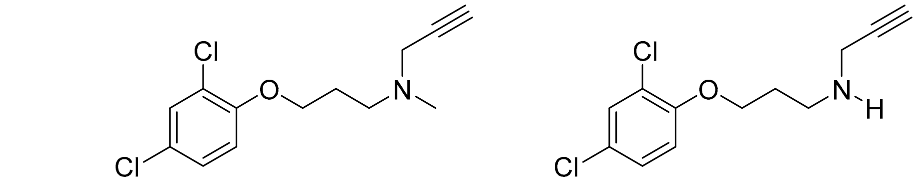

Clorgyline **M5**

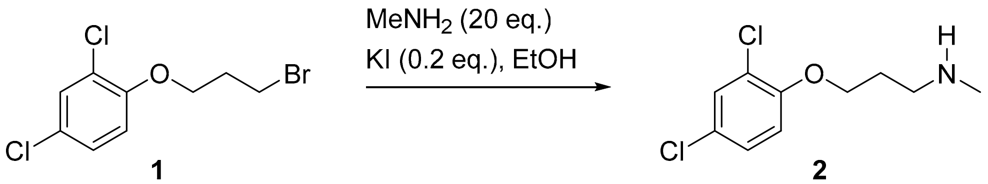

1 **2**

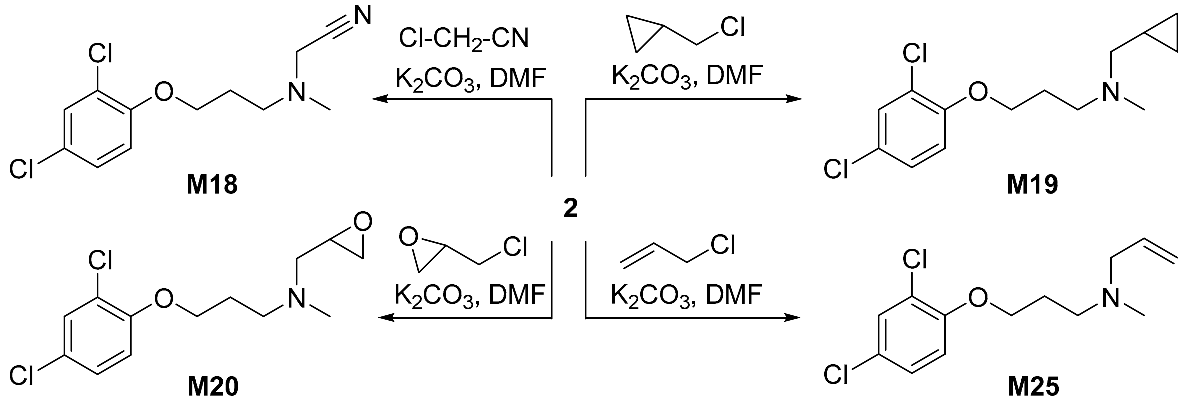

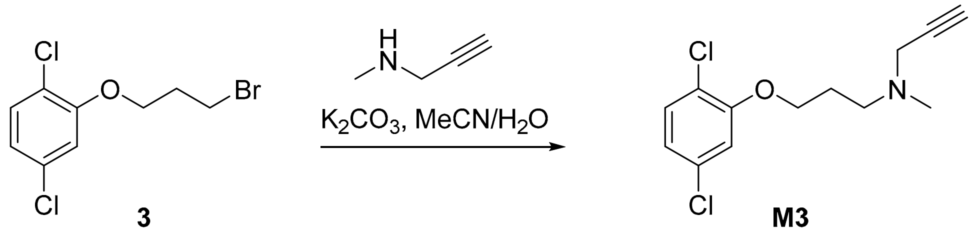

3 **M3**

References:

73. Ohmomo, Y.; Hirata, M.; Murakami, K.; Magata, Y.; Tanaka, C.; Yokoyama, A. Synthesis and characterization of 11C-labeled fluoroclorgyline: a monoamine oxidase A specific inhibitor for positron emission tomography. *Chemical & pharmaceutical bulletin* **1993**, *41*, 1994-1997, doi:10.1248/cpb.41.1994.
74. Leonardi, A.; Barlocco, D.; Montesano, F.; Cignarella, G.; Motta, G.; Testa, R.; Poggesi, E.; Seeber, M.; De Benedetti, P.G.; Fanelli, F. Synthesis, screening, and molecular modeling of new potent and selective antagonists at the alpha 1d adrenergic receptor. *Journal of medicinal chemistry* **2004**, *47*, 1900-1918, doi:10.1021/jm030944+.

Materials and Methods

Flash column chromatography: SiO_2 60 (grain size: 0.0040 - 0.063 mm mesh ASTM) from Merck was used as the stationary phase.

Infrared spectroscopy: ATR spectra were recorded with a Jasco FT / IR-4100 with ATR PRO450-S.

NMR spectra (^{1}H-NMR, ^{13}C-NMR, DEPT, COSY, HMQC, HMBC) spectra were recorded, unless otherwise stated, at room temperature using an Avance III HD 400 MHz Bruker Biospin (400 MHz). ^{1}H-chemical – and ^{13}C chemical shifts are given in δ units relative to the deuterated solvent peak. The chemical shifts are reported in parts per million [ppm] and refer to the δ scalar. Coupling constants *J* are indicated in Hertz [Hz]. NMR spectra were displayed and evaluated using MestReNova x64.

Mass spectrometry: For the measurements of high-resolution mass spectra, electron spray ionization (ESI) and electron impact ionization (EI) were used as methods. EI measurements were performed on a Thermo Q Exactive GC Orbitrap or Finnigan MAT 95 sector field mass spectrometer. The ionization (EI) was conducted at a source temperature of 250 °C and an electron energy of 70 eV. Direct Evaporation Samples (DEP/EI) were heated on a platinum thread from 20 to 1600 °C at a rate of 120 °C/min. Gas chromatography (GC/EI) was performed on a Thermo Trace 1300 gas chromatograph with temperature programmable injector (Q Exactive GC) or on a Varian 3400 with split-splitless injector (MAT 95) and autosampler. Electron Spray Ionization (ESI) measurements were performed on a Thermo Finnigan LTQ FT Ultra Fourier Transform Ion Cyclotron Resonance Mass Spectrometer. Flow Injection Analysis (FIA/ESI) was performed with a Surveyor MS pump at a flow rate of 100 μl/min using 20/80 % or 80/20 % water/acetonitrile as a running agent.

High-pressure liquid chromatography: For the determination of purities, high-pressure liquid chromatography (HPLC) measurements of the compounds were performed on a HP Agilent 1100 HPLC system equipped withan autosampler, a column oven, a quaternary pump system and a UV-VWS detector using a Zorbax eclipse Plus C18 (4.6 x 150 mm, 5 μm) column (injection volume: 2 μL or 10 μL, flow rate: 1 mL/min, 50 °C). The signals were detected at the wave lengths of λ = 210 nm and λ = 254 nm, using the following methods: a) acetonitrile/water 70:30; b) acetonitrile/phosphate buffer 50:50, pH ~ 5.

Syntheses:

3-(2,4-Dichlorophenoxy)-*N*-methylpropan-1-amine (2)

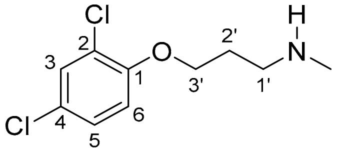

A solution of 1-(3-bromopropoxy)-2,4-dichlorobenzene (**1**) (406 mg, 1.43 mmol, 1.00 eq.) in EtOH, (2.5 mL) was added to a solution of methylamine alcohol (3.13 mL, 28.6 mmol, 33 wt% in ethanol, 20.0 eq.), followed by KI (48.7 mg, 0.293 mmol, 0.205 eq.). The reaction mixture was stirred at room temperature for 1 h 15 min and was then refluxed (85°C) for an additional hour. The mixture was allowed to cool down to room temperature. The solvent was evaporated *in vacuo*. The product was obtained as a white solid and used without further purification for the next step.

^{1}H-NMR (400 MHz, CD_3OD) *δ*/ppm = 7.43 (d, *J* = 2.6 Hz, 1H, 3-H), 7.29 (dd, *J* = 8.9, 2.5 Hz, 1H, 5-H), 7.10 (d, *J* = 8.9 Hz, 1H, 6-H), 4.21 (t, *J* = 5.7 Hz, 2H, 3′-H), 3.26 (t, *J* = 7.2 Hz, 2H, 1′-H), 2.76 (s, 3H, N-CH_3), 2.28 – 2.20 (m, 2H, H-2′). **^{13}C-NMR (101 MHz, CD_3OD)** *δ*/ppm = 154.28 (C-1), 130.7 (C-3), 129.1 (C-5), 127.3 (C-2/C-4), 124.5 (C-2/C-4), 115.8 (C-6), 67.9 (C-3′), 48.4 (C-1′), 34.0 (N-CH_3), 27.0 (C-2′). **IR:** $\tilde{\nu}$ (cm^{-1}) = 2924 (s), 2854 (m), 2790 (s), 2733 (m), 1586 (w), 1568 (w), 1289 (s), 1268 (s), 1256 (s), 1242 (s), 1105 (s), 1061 (s), 1038 (s), 804 (s). **HR-MS (ESI):** 234.04451 $[M+H]^+$ (calcd. for $C_{10}H_{14}{}^{35}Cl_2NO^+$: 234.04470).

2-((3-(2,4-Dichlorophenoxy)propyl)(methyl)amino)acetonitrile (M18)

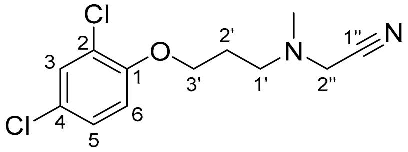

K_2CO_3 (143 mg, 0.865 mmol, 1.99 eq.) was added to a solution of 3-(2,4-dichlorophenoxy)-*N*-methylpropan-1-amine (**2**) (102 mg, 0.434 mmol, 1.00 eq.) in DMF (1 mL). The reaction mixture was stirred for 45 min. at room temperature. At 0 °C, chloroacetonitrile (54.9 µL, 0.867 mmol, 2.00 eq.) was added. The reaction mixture was allowed to warm to room temperature and stirred for 4 h, to give a brown suspension. Water (10 mL) was added and the resulting mixture was extracted with EtOAc (3 x 20 mL). The combined organic phase was dried over Na_2SO_4, filtered and concentrated *in vacuo*. The crude product was purified by flash column chromatography (hexanes/EtOAc = 7:3). **M18** was obtained as a yellow oil. Yield; 54.1 mg (0.20 mmol, 46 %)

^{1}H-NMR (400 MHz, $CDCl_3$) *δ*/ppm = 7.36 (d, *J* = 2.5 Hz, 1H, 3-H), 7.17 (dd, *J* = 8.8, 2.6 Hz, 1H, 5-H), 6.84 (d, *J* = 8.8 Hz, 1H, 6-H), 4.06 (t, *J* = 6.1 Hz, 2H, 3′-H), 3.56 (s, 2H, 2′′-H), 2.70 (t, *J* = 6.9 Hz, 2H, 1′-H), 2.39 (s, 3H, N-CH_3), 1.99 (p, *J* = 6.4 Hz, 2H, 2′-H). **^{13}C-NMR (101 MHz, $CDCl_3$)** *δ*/ppm = 153.4 (C-1), 130.1 (C-3), 127.70 (C-5), 126.0 (C-2/C-4), 123.9 (C-2/C-4), 114.8 (C-1′′), 114.3 (C-6), 66.9 (C-3′), 52.3 (C-1′), 45.6 (C-2′′), 42.0 (N-CH_3), 27.1 (C-2′). **IR:** $\tilde{\nu}$ (cm^{-1}) = 3071 (w), 2953 (m), 2854 (w), 2811 (w), 2231 (w), 2136 (w), 1674 (w), 1586 (w), 1288 (s), 1256 (s), 1103 (s), 1061 (s), 804 (s). **HR-MS (ESI):**

273.05560 [M+H]$^+$ (calcd. for $C_{12}H_{15}{}^{35}Cl_2N_2O^+$: 273.05559). **Purity (HPLC):** >99 % (λ = 210 nm; method a), >99 % (λ = 254 nm; method a).

N-(Cyclopropylmethyl)-3-(2,4-dichlorophenoxy)-*N*-methylpropan-1-amine (M19)

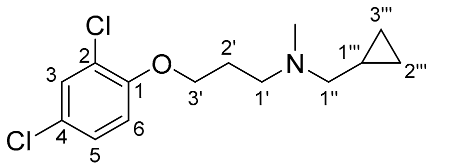

To a solution of 3-(2,4-dichlorophenoxy)-*N*-methylpropan-1-amine (**2**) (109 mg, 0.466 mmol, 1.00 eq.) in DMF (1 mL), K_2CO_3 (153 mg, 0.927 mmol, 1.99 eq.) was added and the mixture was stirred for 45 min. at room temperature. (Chloromethyl)cyclopropane (90.6 µL, 0.930 mmol, 1.99 eq.) was added at 0 °C. The reaction mixture was allowed to warm to room temperature and stirred at this temperature for 1 h. The mixture was then heated to 70 °C for 2 h, cooled to room temperature and stirred for 3 d. The suspension was then refluxed for additional 2 h (70 °C). After cooling, the suspension was diluted with water (20 mL) and extracted with EtOAc (3x 30 mL). The organic layer was dried over Na_2SO_4, filtered and the solvent removed *in vacuo*. The crude product was purified by flash column chromatography on silica (EtOAc/1.0 % triethylamine = 10:0.1). **M19** was obtained as a yellow oil. Yield: 41.1 mg (0.14 mmol, 31 %).

^{1}H-NMR (400 MHz, $CDCl_3$) δ/ppm = 7.35 (d, J = 2.6 Hz, 1H, 3-H), 7.16 (dd, J = 8.8, 2.6 Hz, 1H, 5-H), 6.86 (d, J = 8.8 Hz, 1H, 6-H), 4.07 (t, J = 6.3 Hz, 2H, 3′-H), 2.61 (t, J = 7.1 Hz, 2H, 1′-H), 2.32 (s, 3H, N-CH_3), 2.26 (d, J = 6.6 Hz, 2H, 1′′-H), 1.99 (p, J = 6.6 Hz, 2H, 2′-H), 0.93 – 0.78 (m, 1H, 1′′′-H), 0.56 – 0.44 (m, 2H, 2′′′-H, 3′′′-H), 0.14 – 0.03 (m, 2H, 2′′′-H, 3′′′-H). **^{13}C-NMR (101 MHz, $CDCl_3$)** δ = 153.6 (C-1), 130.0 (C-3), 127.6 (C-5), 125.6 (C-2/C-4), 123.9(C-2/C-4), 114.2 (C-6), 67.0 (C-3′), 63.0 (C-1′′), 54.0 (C-1′), 42.6 (N-CH_3), 27.2 (C-2′), 9.04 (C-1′′′), 4.06 (C-2′′′, C-3′′′) ppm. **IR:** $\tilde{\nu}$ (cm^{-1}) = 3076 (w), 3000 (m), 2948 (m), 2879 (m), 2843 (m), 2795 (m), 2771 (m), 1587 (m), 1570 (w), 1265 (s), 1104 (s), 1062 (s), 1046 (m), 1017 (s), 803 (s). **HR-MS (ESI):** 288.09153 [M+H]$^+$ (calcd. for $C_{14}H_{20}{}^{35}Cl_2NO^+$: 288.09165). **Purity (HPLC):** >99 % (λ = 210 nm; method b), >99 % (λ = 254 nm; method b).

3-(2,4-Dichlorophenoxy)-*N*-methyl-*N*-(oxiran-2-ylmethyl)propan-1-amine (M20)

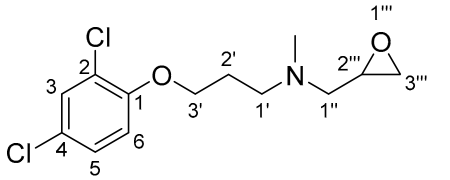

A suspension of 3-(2,4-dichlorophenoxy)-*N*-methylpropan-1-amine (**4**) (110 mg, 0.47 mmol, 1.00 eq.) and K_2CO_3 (156 mg, 0.945 mmol, 2.02 eq.) in DMF (1 mL) was stirred for 45 min. at room temperature. Epichlorohydrine (73.6 µL, 0.938 mmol, 2.00 eq.) was added at 0 °C. The reaction mixture was allowed to warm to room temperature and stirred for 1 h. The mixture was heated to 70 °C for 2 h. The mixture was then allowed to cool to room temperature and was stirred for additional 3 d. The mixture was diluted with water (20 mL) and extracted with EtOAc (3 x 30 mL). The organic layer was dried over Na_2SO_4, filtered and the volatiles removed *in vacuo*. The crude product was purified by column

chromatography (DCM/MeOH = 99:1) and **M20** obtained as a yellow oil. Yield: 14.9 mg (0.051 mmol, 11 %).

^{1}H-NMR (400 MHz, $CDCl_3$) δ/ppm = 7.35 (d, *J* = 2.5 Hz, 1H, 3-H), 7.16 (dd, *J* = 8.8, 2.6 Hz, 1H, 5-H), 6.86 (d, *J* = 8.8 Hz, 1H, 6-H), 4.08 (td, *J* = 6.3, 1.5 Hz, 2H, 3′-H), 3.06 (dtd, *J* = 6.5, 3.8, 2.6 Hz, 1H, 2′′′-H), 2.77 (dd, *J* = 5.0, 4.0 Hz, 1H, 1′′-H), 2.72 (dd, *J* = 13.4, 3.6 Hz, 1H, 3′′′-H), 2.65 (td, *J* = 6.9, 4.4 Hz, 2H, 1′-H), 2.49 (dd, *J* = 5.0, 2.7 Hz, 1H, 1′′-H), 2.36 (dd, *J* = 13.4, 6.5 Hz, 1H, 3′′′-H), 2.35 (s, 3H, N-CH_3), 2.00 (p, *J* = 6.5 Hz, 2H, 2′-H). **^{13}C-NMR (101 MHz, $CDCl_3$)** δ/ppm = 153.6 (C-1), 130.1 (C-3), 127.7 (C-5), 125.7 (C-2/C-4), 123.9 (C-2/C-4), 114.3 (C-6), 67.6 (C-3′), 60.4 (C-3′′′), 54.3 (C-1′), 50.8 (C-2′′′), 45.3 (C-1′′), 43.0 (N-CH_3), 27.1 (C-2′). **IR:** $\tilde{\nu}$ (cm^{-1}) = 3047 (w), 2925 (m), 2848 (m), 2790 (m), 1586 (w), 1571 (w), 1256 (s), 1103 (s), 1061 (s), 803 (s). **HR-MS (ESI):** 290.07095 $[M+H]^+$ (calcd. for $C_{13}H_{18}{}^{35}Cl_2NO_2{}^+$: 290.07091). **Purity (HPLC):** >99 % (λ = 210 nm; method b), >99 % (λ = 254 nm; method b).

***N*-(3-(2,4-Dichlorophenoxy)propyl)-*N*-methylprop-2-en-1-amine (M25)**

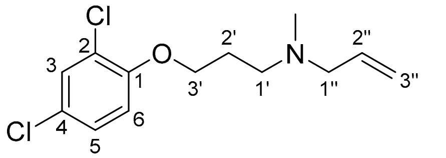

3-(2,4-Dichlorophenoxy)-*N*-methylpropan-1-amine (**2**) (77.1 mg, 0.329 mmol, 1.00 eq.) and K_2CO_3 (109 mg, 0.658 mmol, 2.00 eq.) in DMF (0.7 mL), were stirred for 45 min. at room temperature. Allyl chloride (53.7 µL, 0.659 mmol, 2.00 eq.) was added at 0 °C. The reaction mixture was allowed to warm to room temperature and stirred for 6 h, to give a colourless suspension. Water (10 mL) was added and the mixture was extracted with EtOAc (4 x 20 mL). The organic layer was dried over Na_2SO_4, filtered and concentrated *in vacuo*. The crude product was purified by flash column chromatography on silica (EtOAc/1.0 % triethylamine = 10:0.1). **M25** was obtained as a yellow oil. Yield: 30.6 mg (0.11 mmol, 34 %).

^{1}H-NMR (400 MHz, $CDCl_3$) δ/ppm = 7.35 (d, *J* = 2.6 Hz, 1H, 3-H), 7.16 (dd, *J* = 8.8, 2.6 Hz, 1H, 5-H), 6.85 (d, *J* = 8.8 Hz, 1H, 6-H), 5.84 (ddt, *J* = 16.8, 10.2, 6.5 Hz, 1H, 2′′-H), 5.21 – 5.14 (m, 1H, 3′′-H), 5.12 (ddt, *J* = 10.2, 2.2, 1.2 Hz, 1H, 3′′-H), 4.06 (t, *J* = 6.3 Hz, 2H, 3′-H), 3.02 (dt, *J* = 6.6, 1.3 Hz, 2H, 1′′-H), 2.55 (t, *J* = 7.1 Hz, 2H, 1′-H), 2.24 (s, 3H, N-CH_3), 1.99 (p, *J* = 6.5 Hz, 2H, 2′-H). **^{13}C-NMR (101 MHz, $CDCl_3$)** δ/ppm = 153.58 (C-1), 135.82 (C-2′′), 130.05 (C-3), 127.63 (C-5), 125.64 (C-2/C-4), 123.88 (C-2/C-4), 117.63 (C-3′′), 114.25 (C-6), 67.78 (C-3′), 61.17 (C-1′′), 53.58 (C-1′), 42.27 (N-CH_3), 27.22 (C-2′). **IR:** $\tilde{\nu}$ (cm^{-1}) = 3075 (w), 2950 (m), 2878 (m), 2844 (m), 2793 (m), 1586 (m), 1389 (m), 1265 (s), 1256 (s), 1104 (s), 1061 (s), 920 (s), 803 (s). **HR-MS (ESI):** $[M+H]^+$ (calcd.). 274.07592 $[M+H]^+$ (calcd. for $C_{13}H_{18}{}^{35}Cl_2NO^+$: 274.07600). **Purity (HPLC):** >99 % (λ = 210 nm; method b), >99 % (λ = 254 nm; method b)-

N-(3-(2,5-Dichlorophenoxy)propyl)-N-methylprop-2-yn-1-amine (M3)

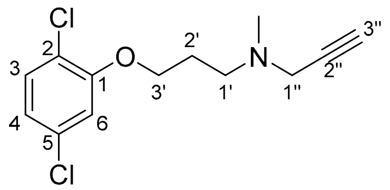

To a solution of 2-(3-bromopropoxy)-1,4-dichlorobenzene (**3**) (475 mg, 1.67 mmol, 1.00 eq.) in acetonitrile (5 mL), a solution of potassium carbonate (253 mg, 1.83 mmol, 1.09 eq.) in water (0.5 mL) and *N*-methylpropargylamine (0.28 mL, 3.3 mmol, 2.0 eq.) were added. The reaction mixture was stirred at room temperature for 18 h. The acetonitrile-water phase was decanted and evaporated *in vacuo*. The residue was taken up with ether (30 mL) and washed with water (4 x 20 mL). The organic phase was dried over sodium sulfate, filtered and the solvent was evaporated *in vacuo*. The crude product was purified by flash column chromatography on silica (hexanes/EtOAc/0.5 % triethylamine = 5:5:0.05). **M3** was obtained as a yellow oil. Yield: 300 mg (1.10 mmol, 66 %).

^{1}H-NMR (400 MHz, CDCl$_3$) δ/ppm = 7.26 (d, J = 8.5 Hz, 1H, 3-H), 6.93 (d, J = 2.3 Hz, 1H, 6-H), 6.86 (dd, J = 8.4, 2.3 Hz, 1H, 4-H), 4.07 (t, J = 6.3 Hz, 2H, 3′-H), 3.37 (d, J = 2.4 Hz, 2H, 1′′-H), 2.64 (t, J = 7.0 Hz, 2H, 1′-H), 2.33 (s, 3H, N-CH$_3$), 2.22 (t, J = 2.4 Hz, 1H, 3′′-H), 1.99 (p, J = 6.5 Hz, 2H, 2′-H). **^{13}C-NMR (101 MHz, CDCl$_3$)** δ/ppm = 155.1 (C-1), 133.1 (C-5), 130.8 (C-3), 121.5 (C-2), 121.3 (C-4), 114.0 (C-6), 78.6 (C-2′′), 73.3 (C-3′′), 67.5 (C-3′), 52.1 (C-1′), 45.9 (C-1′′), 41.9 (N-CH$_3$), 27.2 (C-2′). **IR:** $\tilde{\nu}$ (cm^{-1}) = 3300 (m), 2945 (m), 2842 (w), 2802 (w), 2150 (w), 2100 (w), 1262 (s), 1132 (s), 1061 (s), 890 (m), 837 (m), 801 (s), 717 (m). **HR-MS (EI):** 271.0520 [M]$^{\bullet+}$ (calcd. for $C_{13}H_{15}Cl_2NO^{\bullet+}$: 271.0525). **Purity (HPLC):** >99 % (λ = 210 nm; method a), >99 % (λ = 254 nm; method a).

Table S1. Zone of inhibition ratio in disk diffusion screen.

Organism	Strain	Antifungal [nmol/disk]	Modulator [50nmol/disk]						
			CLO	M3	M5	M18	M19	M20	M25
S. cerevisiae	ADΔΔ	POS [0.05]	**1.3**	**1.3**	**1.3**	**1.2**	0.6	0.4	**1.8**
		VRC [0.05]	**1.2**	**1.1**	**1.1**	1.0	0.6	0.4	**1.3**
		FLC [3]	**1.8**	**1.7**	**1.7**	**1.5**	0.7	0.5	**1.7**
S. cerevisiae	CaMdr1A	POS [0.05]	**1.2**	**1.1**	**1.2**	**1.1**	0.4	0.3	**1.2**
		VRC [0.75]	**2.0**	**1.7**	**1.7**	**1.3**	0.8	0.4	**2.0**
		FLC [50]	**1.9**	**1.8**	**1.8**	1.0	**1.2**	**1.1**	**1.9**
S. cerevisiae	CaCdr1B	POS [45]	**1.8**	**1.7**	**1.8**	**1.5**	**1.2**	**1.2**	**1.7**
		VRC [12.5]	**1.8**	**1.9**	**1.9**	**1.7**	**1.3**	**1.3**	**1.7**
		FLC [50]	1.0	1.0	1.0	1.0	1.0	1.0	1.0
C. auris	CBS10913T	POS [0.3]	1.0	1.0	1.0	0.9	1.0	1.0	1.0
		VRC [6.25]	1.0	1.0	1.0	0.9	1.0	1.0	1.0
		FLC [50]	1.0	1.0	1.0	1.0	1.0	1.0	1.0
C. auris	AR0389	POS [0.3]	1.0	1.0	0.9	0.9	**1.3**	**1.3**	**1.2**
		VRC [25]	0.7	0.7	0.0	0.9	**1.1**	1.0	1.0
		FLC [50]	1.0	1.0	1.0	1.0	1.0	1.0	1.0

Synergistic effect of compounds (zone of inhibition ratio >1) is highlighted in bold on a green background. Different concentrations of antifungal and modulator were screened and the concentration of azole giving an inhibition zone of ~0.5 mm radius was chosen for POS and VRC. While the *S. cerevisiae* strains gave suitable sized inhibition zones with FLC at the indicated concentrations, neither *C. auris* strain gave an inhibition zone with the highest amount of FLC added on disk. The inhibition zone with the azole drug alone was compared with inhibition zone obtained in combination with 50 nmol/disk of modulator.

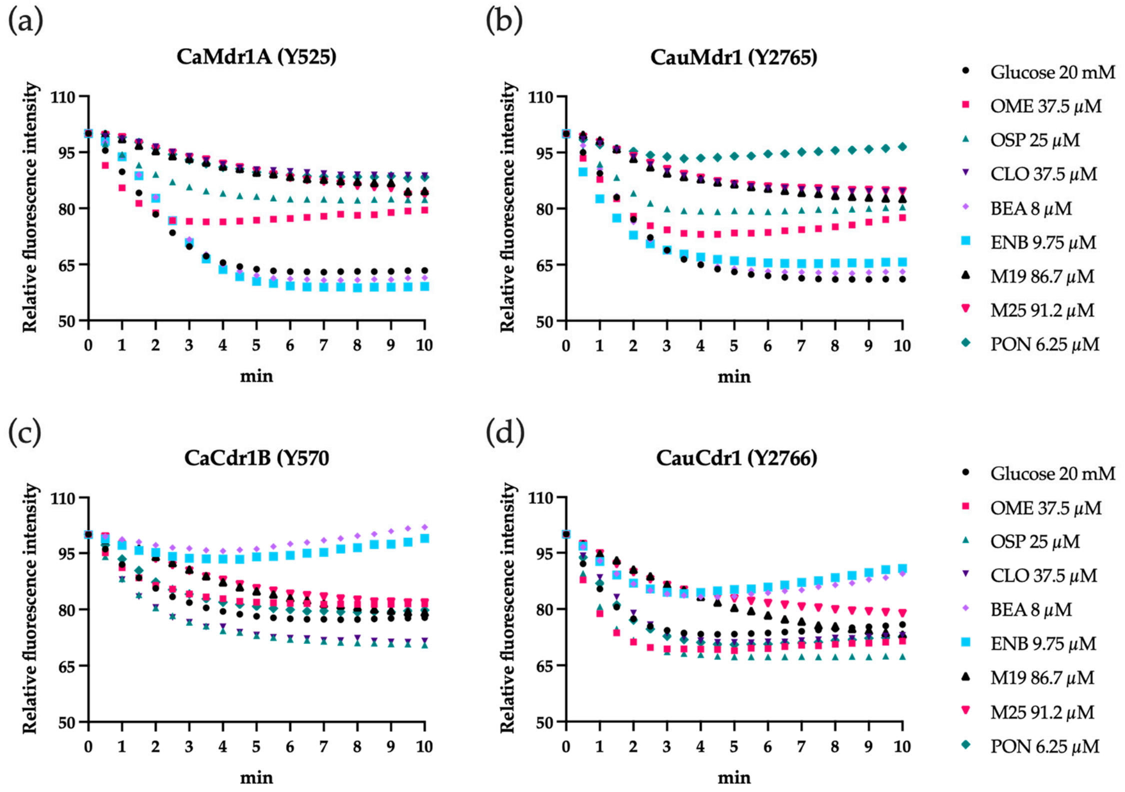

Figure S2: Effects of efflux modulators on glucose-dependent Nile Red efflux by recombinant strains. Shown is mean of three biological replicates in technical duplicates. SD is not shown for better overview.

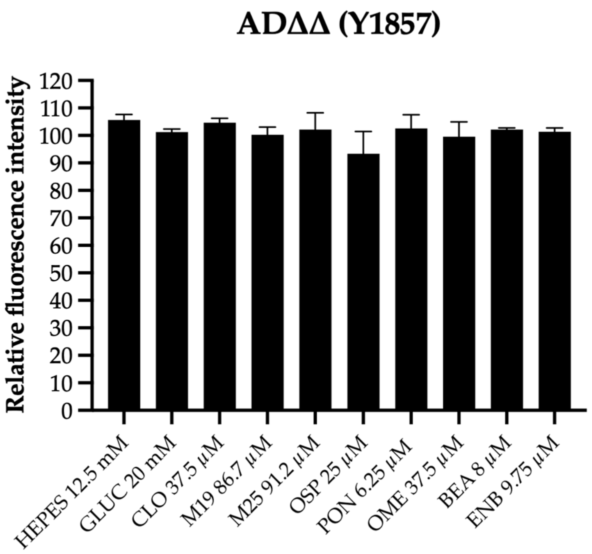

Figure S3. Glucose-dependent Nile Red efflux by the control strain ADΔΔ. After ten minutes incubation the steady state Nile red efflux remained at 100%. The experiment was carried out in three biological replicates (n=3) with technical duplicates. Bars represents standard deviation.

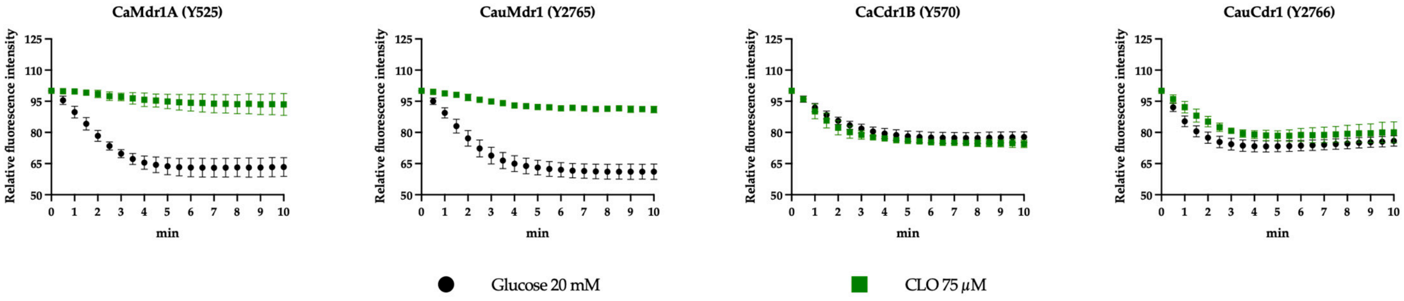

Figure S4: Effect of 75 μM CLO on glucose-dependent Nile Red efflux by recombinant strains. Shown is mean of three biological replicates (n=3) in technical duplicates with bars representing standard deviation.

Table S2. Checkerboard susceptibility assays of recombinant strains overexpressing CauCdr1 and CauMdr1 for azole drugs in combination with modulators.

		MIC$_{80}$ [µM]							
		Alone		Combination		FIC			
Strain	AF/MOD	AF	MOD	AF	MOD	AF	MOD	FICI	Effect
CauMdr1 Y2765	POS/CLO	0.13	98.7	0.03	33.9	0.20	0.34	0.55	NI
	POS/M19	0.16	86.7	0.05	25.3	0.29	0.29	0.58	NI
	POS/M25	0.09	182	0.01	39.8	0.13	0.22	0.34	SYN
	POS/PON	0.25	6.15	0.09	2.31	0.36	0.38	0.74	NI
	POS/OSP	0.09	≥370	0.05	2.89	0.50	0.01	0.51	NI
	POS/OME	0.23	400	0.09	37.5	0.40	0.09	0.49	SYN
	POS/RAB	0.08	208	0.02	34.7	0.25	0.17	0.42	SYN
	POS/ENB	0.25	14.4	0.13	1.65	0.51	0.11	0.63	NI
	POS/BEA	0.25	7.50	0.12	0.86	0.47	0.11	0.58	NI
	VRC/CLO	12.5	98.7	2.08	24.7	0.17	0.25	0.42	SYN
	VRC/M19	7.81	86.7	1.56	32.5	0.20	0.38	0.58	NI
	VRC/M25	6.25	182	1.56	45.5	0.25	0.25	0.50	SYN
	VRC/PON	8.33	7.71	1.82	1.16	0.22	0.15	0.37	SYN
	VRC/OSP	7.71	≥370	6.17	3.85	0.80	0.01	0.81	NI
	VRC/OME	14.6	400	4.17	41.7	0.33	0.10	0.43	SYN
	VRC/RAB	9.25	208	1.35	77.2	0.15	0.37	0.52	NI
	VRC/ENB	8.33	14.4	8.33	0.26	1.00	0.02	1.02	NI
	VRC/BEA	5.21	7.50	0.05	5.90	0.01	0.79	0.80	NI
	ISA/CLO	0.13	55.5	0.02	13.9	0.15	0.25	0.40	SYN
	ISA/M19	0.13	86.4	0.02	32.4	0.17	0.38	0.54	NI
	ISA/M25	0.13	133.3	0.02	33.3	0.17	0.25	0.42	SYN
	ISA/OSP	0.10	≥370	0.06	2.89	0.60	0.01	0.61	NI
	ISA/OME	0.05	400	0.01	50.0	0.14	0.13	0.26	SYN
CauCdr1 Y2766	POS/CLO	1.75	123	0.10	49.3	0.06	0.40	0.46	SYN
	POS/M19	0.98	108	0.22	19.0	0.23	0.18	0.40	SYN
	POS/M25	0.49	182	0.09	39.8	0.19	0.22	0.41	SYN
	POS/PON	1.00	50.0	0.75	1.15	0.75	0.02	0.77	NI
	POS/OSP	0.75	≥370	0.75	2.89	1.00	0.01	1.01	NI
	POS/OME	0.25	1100	0.06	57.3	0.25	0.05	0.30	SYN
	POS/RAB	1.25	≥3750	0.75	31.3	0.60	0.01	0.61	NI
	POS/ENB	1.25	9.13	0.27	0.90	0.21	0.10	0.31	SYN
	POS/BEA	1.13	11.9	0.08	1.66	0.07	0.14	0.21	SYN
	VRC/CLO	25.0	123	3.65	49.3	0.15	0.40	0.55	NI
	VRC/M19	21.9	130	5.47	21.7	0.25	0.17	0.42	SYN
	VRC/M25	12.5	182	6.25	22.8	0.50	0.13	0.63	NI
	VRC/PON	25.0	50.0	20.8	0.62	0.83	0.01	0.85	NI
	VRC/OSP	18.5	≥370	18.5	2.89	1.00	0.01	1.01	NI
	VRC/OME	9.25	1100	2.31	275	0.25	0.25	0.50	SYN
	VRC/RAB	18.5	≥3750	18.5	1.45	1.00	0.0004	1.00	NI

VRC/ENB	25.0	13.9	1.30	1.50	0.05	0.11	0.16	SYN
VRC/BEA	20.8	9.83	1.30	1.48	0.06	0.15	0.21	SYN
ISA/CLO	14.5	111	2.06	46.3	0.14	0.42	0.56	NI
ISA/M19	13.7	130	1.96	54.0	0.14	0.42	0.56	NI
ISA/M25	13.7	267	2.17	111	0.16	0.42	0.58	NI
ISA/OSP	15.4	≥370	15.42	2.89	1.00	0.01	1.01	NI
ISA/OME	2.30	1470	1.16	34.4	0.50	0.02	0.53	NI

Average values of at least three biological replicates (n=3), AF antifungal, MOD modulator, SYN Synergy, NI no interaction, ≥ MIC equal or higher than shown, solubility limit reached. ISA not tested in combination with PON, RAB, ENB, or BEA, respectively.

Table S3. Checkerboard susceptibility assays of recombinant strains overexpressing CaCdr1 and CaMdr1 for azole drugs in combination with modulators.

		MIC$_{80}$ [µM]							
		Alone		Combination		FIC			
Strain	AF/MOD	AF	MOD	AF	MOD	AF	MOD	FICI	Effect
CaMdr1A Y525	POS/CLO	0.07	123	0.001	98.7	0.01	0.80	0.81	NI
	POS/M19	0.17	86.7	0.05	22.1	0.33	0.26	0.58	NI
	POS/M25	0.11	151	0.03	37.8	0.25	0.25	0.50	SYN
	POS/PON	0.06	8.09	0.03	3.47	0.50	0.43	0.93	NI
	POS/OME	0.17	533	0.05	167	0.30	0.31	0.61	NI
	POS/ENB	0.15	21.6	0.08	0.23	0.50	0.01	0.51	NI
	POS/BEA	0.08	11.8	0.04	1.48	0.50	0.13	0.63	NI
	VRC/CLO	3.33	123	0.67	15.4	0.20	0.13	0.33	SYN
	VRC/M19	2.00	86.7	1.00	10.8	0.50	0.13	0.63	NI
	VRC/M25	1.20	151	0.30	30.3	0.25	0.20	0.45	SYN
	VRC/PON	4.00	9.25	0.50	0.77	0.13	0.08	0.21	SYN
	VRC/OME	2.00	667	1.00	100	0.50	0.15	0.65	NI
	VRC/ENB	3.00	21.6	2.00	0.34	0.67	0.02	0.68	NI
	VRC/BEA	3.00	11.8	2.00	0.18	0.67	0.02	0.68	NI
	ISA/CLO	0.33	92.5	0.07	13.9	0.22	0.15	0.37	SYN
	ISA/M19	0.21	130	0.04	32.4	0.20	0.25	0.45	SYN
	ISA/M25	0.29	267	0.04	33.3	0.14	0.13	0.27	SYN
	ISA/OME	0.05	667	0.003	333	0.05	0.50	0.55	NI
CaCdr1B Y570	POS/CLO	≥60.0	150	2.81	46.3	0.05	0.31	0.36	SYN
	POS/M19	≥60.0	86.7	10.3	10.8	0.17	0.13	0.30	SYN
	POS/M25	≥60.0	182	7.50	37.9	0.13	0.21	0.33	SYN
	POS/PON	≥60.0	55.2	9.50	19.5	0.16	0.35	0.51	NI
	POS/OME	7.50	≥4400	3.75	34.4	0.50	0.01	0.51	NI
	POS/ENB	≥60.0	5.40	3.75	0.23	0.06	0.04	0.10	SYN
	POS/BEA	≥60.0	11.8	1.28	0.61	0.02	0.05	0.07	SYN
	VRC/CLO	37.0	148	7.71	43.2	0.21	0.29	0.50	SYN
	VRC/M19	41.7	86.7	16.7	18.1	0.40	0.21	0.61	NI
	VRC/M25	41.7	182	20.8	30.3	0.50	0.17	0.67	NI
	VRC/PON	37.0	55.2	37.0	0.58	1.00	0.01	1.01	NI
	VRC/OME	37.0	≥4400	9.25	1100	0.25	0.25	0.50	SYN
	VRC/ENB	37.0	5.40	1.54	0.45	0.04	0.08	0.13	SYN
	VRC/BEA	49.3	9.83	1.64	0.98	0.03	0.10	0.13	SYN
	ISA/CLO	≥100	137	10.8	55.5	0.11	0.41	0.51	NI
	ISA/M19	≥100	130	37.0	21.6	0.37	0.17	0.54	NI
	ISA/M25	≥100	267	27.8	66.7	0.28	0.25	0.53	NI
	ISA/OME	37.0	≥4400	9.25	550	0.25	0.13	0.38	SYN

Average values of at least three biological replicates (n=3), AF antifungal, MOD modulator, SYN synergy, NI no interaction, ≥ solubility limit of compound reached. OSP and RAB not tested. ISA only in combination with OME and CLO with its analogs tested.

Table S2. Checkerboard susceptibility assays for recombinant strains overexpressing CauErg11 for azole drugs in combination with modulators.

		MIC_{80} [µM]							
		Alone		Combination		FIC			
Strain	AF/MOD	AF	MOD	AF	MOD	AF	MOD	FICI	Effect
CauErg11 Y2767	POS/CLO	0.21	149	0.13	1.54	0.63	0.01	0.64	NI
	POS/M19	0.30	86.7	0.05	43.4	0.17	0.50	0.67	NI
	POS/M25	0.30	137	0.09	60.7	0.30	0.44	0.75	NI
	POS/PON	0.20	9.25	0.001	9.25	0.004	1.00	1.00	NI
	POS/OSP	0.10	≥370	0.05	2.89	0.50	0.01	0.51	NI
	POS/OME	0.13	66.7	0.004	66.7	0.03	1.00	1.03	NI
	POS/RAB	0.13	208	0.06	42.4	0.45	0.20	0.65	NI
	POS/ENB	0.31	15.7	0.16	6.08	0.51	0.39	0.89	NI
	POS/BEA	0.41	15.7	0.16	4.98	0.38	0.32	0.70	NI
	VRC/CLO	0.25	136	0.10	43.2	0.42	0.32	0.73	NI
	VRC /M19	0.21	86.7	0.10	28.9	0.50	0.33	0.83	NI
	VRC /M25	0.21	137	0.13	45.5	0.60	0.33	0.93	NI
	VRC/PON	0.21	9.25	0.004	9.25	0.02	1.00	1.02	NI
	VRC /OSP	0.17	≥370	0.13	2.89	0.75	0.01	0.76	NI
	VRC /OME	0.13	66.7	0.004	66.7	0.03	1.00	1.03	NI
	VRC/RAB	0.25	208	0.25	1.45	1.00	0.01	1.01	NI
	VRC /ENB	0.13	15.0	0.08	4.91	0.68	0.33	1.00	NI
	VRC/BEA	0.17	19.7	0.17	0.15	1.00	0.01	1.01	NI
	ISA/CLO	0.02	111	0.01	11.6	0.60	0.10	0.70	NI
	ISA/M19	0.02	64.8	0.004	48.6	0.21	0.75	0.96	NI
	ISA /M25	0.02	133	0.01	55.5	0.63	0.42	1.04	NI
	ISA /OSP	0.03	≥370	0.03	2.89	0.75	0.01	0.76	NI
	ISA /OME	0.02	66.7	0.0004	66.7	0.02	1.00	1.02	NI

Average values of at least three biological replicates (n=3), AF antifungal, MOD modulator, SYN synergy, NI no interaction, ≥ solubility limit of compound reached. ISA in combination with PON, RAB, ENB, BEA, respectively, not tested.

Table S3. Checkerboard susceptibilities assays for *C. auris* clinical isolates for azole drugs in combination with CLO, M19, and M25.

		MIC80 [mg/L]							
		Alone		Combination		FIC			
Strain	AF/MOD	AF	MOD	AF	MOD	AF	MOD	FICI	Effect
CBS12875 clade I	POS/CLO	0.10	≥150	0.05	4.43	0.49	0.03	0.51	NI
	POS/M19	0.13	≥150	0.01	6.25	0.06	0.04	0.10	SYN
	POS/M25	0.10	≥150	0.03	5.21	0.25	0.03	0.28	SYN
	VRC/CLO	4.00	≥150	4.00	0.39	1.00	0.00	1.00	NI
	VRC/M19	4.00	≥150	0.83	6.25	0.21	0.04	0.25	SYN
	VRC/M25	10.7	≥150	2.67	4.69	0.25	0.03	0.28	SYN
	ITC/CLO	0.42	≥150	0.21	0.91	0.50	0.01	0.51	NI
	ITC/M19	1.00	≥150	0.17	5.21	0.17	0.03	0.20	SYN
	VT-1161/CLO	8.33	≥150	6.20	4.43	0.74	0.03	0.77	NI
	VT-1161/M19	10.4	≥150	3.13	4.17	0.30	0.03	0.33	SYN
	MFG/CLO	0.21	≥150	0.29	8.56	1.40	0.06	1.46	NI
	MFG/M19	0.25	≥150	0.50	0.39	2.00	0.00	2.00	NI
AR0389 clade I	POS/CLO	0.06	≥150	0.03	0.78	0.49	0.01	0.50	SYN
	POS/M19	0.05	≥150	0.01	1.56	0.13	0.01	0.14	SYN
	POS/M25	0.21	≥150	0.02	6.25	0.08	0.04	0.12	SYN
	VRC/CLO	5.33	≥150	4.00	0.39	0.75	0.003	0.75	NI
	VRC/M19	4.00	≥150	1.00	4.17	0.25	0.03	0.28	SYN
	VRC/M25	16.0	≥150	3.33	5.21	0.21	0.03	0.24	SYN
	ITC/CLO	0.33	≥150	0.17	4.30	0.51	0.03	0.53	NI
	ITC/M19	0.67	≥150	0.17	4.69	0.25	0.03	0.28	SYN
	VT-1161/CLO	10.4	≥150	10.4	0.39	1.00	0.003	1.00	NI
	VT-1161/M19	10.4	≥150	2.60	4.17	0.25	0.03	0.28	SYN
	MFG/CLO	0.25	≥150	0.29	1.33	1.17	0.01	1.18	NI
	MFG/M19	0.25	≥150	0.92	0.39	3.67	0.003	3.67	NI
CBS10913^T clade II	POS/CLO	0.02	75.0	0.01	25.0	0.50	0.33	0.83	NI
	POS/M19	0.01	≥150	0.001	12.5	0.13	0.08	0.21	SYN
	POS/M25	0.06	≥150	0.02	13.5	0.38	0.09	0.47	SYN
	VRC/CLO	0.02	75.0	0.02	0.39	1.00	0.01	1.01	NI
	VRC/M19	0.02	≥150	0.01	12.5	0.53	0.08	0.62	NI
	VRC/M25	0.17	≥150	0.07	7.29	0.40	0.05	0.45	SYN
	ITC/CLO	0.03	75.0	0.02	0.39	0.60	0.01	0.61	NI
	ITC/M19	0.03	≥150	0.01	4.69	0.27	0.03	0.30	SYN
	VT-1161/CLO	0.08	75.0	0.06	0.39	0.75	0.01	0.76	NI
	VT-1161/M19	0.08	≥150	0.02	3.13	0.19	0.02	0.21	SYN
	MFG/CLO	0.03	75.0	0.03	0.39	1.20	0.01	1.21	NI
	MFG/M19	0.04	≥150	0.03	4.30	0.75	0.03	0.78	NI

Average values of at least three biological replicates (n=3), AF antifungal, MOD modulator, SYN synergy, NI no interaction, ≥ solubility limit of compound.

Table S4. Checkerboard susceptibilities assays for *C. albicans* clinical isolates for azole drugs in combination with M19 and M25.

		MIC80 [µM]				FIC			
		Alone		Combination					
Strain	AF/MOD	AF	MOD	AF	MOD	AF	MOD	FICI	Effect
C. albicans TL1	POS/M19	0.05	≥520	0.06	1.35	1.20	0.003	1.20	NI
	POS/M25	0.05	≥547	0.05	1.42	1.00	0.003	1.00	NI
	VRC/M19	0.03	≥520	0.04	1.35	1.33	0.003	1.34	NI
	VRC/M25	0.04	≥547	0.03	1.42	0.75	0.003	0.75	NI
C. albicans TL3	POS/M19	0.33	≥520	0.26	7.68	0.80	0.01	0.81	NI
	POS/M25	0.52	≥547	0.20	76.0	0.38	0.14	0.51	NI
	VRC/M19	0.78	≥520	0.78	2.26	1.00	0.004	1.00	NI
	VRC/M25	1.04	≥547	1.56	1.42	1.50	0.003	1.50	NI
C. albicans SGY243	POS/M19	0.06	260	0.06	1.35	1.00	0.01	1.01	NI
	POS/M25	0.07	228	0.07	1.90	1.00	0.01	1.01	NI
	VRC/M19	0.03	260	0.03	1.35	1.20	0.01	1.21	NI
	VRC/M25	0.03	228	0.03	1.42	1.00	0.01	1.01	NI
C. albicans FR2	POS/M19	0.19	130	0.13	22.7	0.71	0.17	0.88	NI
	POS/M25	0.16	274	0.16	1.42	1.00	0.01	1.01	NI
	VRC/M19	0.13	130	0.13	1.38	1.00	0.01	1.01	NI
	VRC/M25	0.13	274	0.08	2.85	0.67	0.01	0.68	NI

Average values of at least three biological replicates (n=3), AF antifungal, MOD modulator, SYN synergy, NI no interaction, ≥ solubility limit of compound.
